# Maneuvering Charge Transport via Insulating Polymer Interface for Steering Photoredox Catalysis

**DOI:** 10.1002/advs.202507670

**Published:** 2025-07-28

**Authors:** Qiao‐Ling Mo, Rui Xiong, Bo‐Yuan Ning, Peng Su, Qing Chen, Jun‐Hao Dong, Bai‐Sheng Sa, Jing‐ying Zheng, Yue Wu, Fang‐Xing Xiao

**Affiliations:** ^1^ Center of Analysis and Testing Nanchang University 999 Xuefu Avenue Nanchang Jiangxi Province 330031 P. R. China; ^2^ College of Materials Science and Engineering Fuzhou University New Campus, Fujian Province Minhou 350108 P. R. China; ^3^ State Key Laboratory of Structural Chemistry Fujian Institute of Research on the Structure of Matter Chinese Academy of Sciences Fuzhou Fujian 350002 P. R. China

**Keywords:** charge transfer, non‐conjugated polymer, photocatalytic CO_2_ reduction, polyelectrolyte, selective organic transformation

## Abstract

Maneuvering precise and tunable charge transportation has remained the core issue of photocatalysis, but meets with limited success owing to the ultra‐fast charge recombination rate, scarcity of applicable co‐catalysts, and difficulty in customizing spatially separated carrier transport pathways. Although co‐catalyst engineering affords a convenient strategy to dominate spatial charge migration to the ideal active sites, the conventional co‐catalyst modification strategy fails to exquisitely mediate the interface between co‐catalyst and semiconductor matrix, along with tedious synthesis procedures. Herein, an insulating polyelectrolyte (NCP), poly(diallyldimethylammonium chloride), is uniformly and seamlessly coated on the transition metal chalcogenides (TMCs) substrates via a facile electrostatic self‐assembly approach and strategically serves as the highly efficient catalytic active sites for stimulating multifarious photoredox catalysis, including selective organic transformation and CO_2_ reduction. The crucial roles of such NCP are unambiguously unraveled via comprehensive experimental and theoretical investigations, which include increasing reactant adsorption, providing active sites, and most importantly, boosting interfacial charge transfer rate. The electron‐withdrawing capability of NCP fosters the effective charge separation over TMCs, leading to the concomitantly improved and stable photocatalytic activities toward aromatic nitro compounds reduction and CO_2_‐to‐syngas conversion under visible light. Our work could strengthen our fundamental understanding of the generic unanticipated charge transport characteristics of insulating polymers for solar energy conversion.

## Graphical abstract

Non‐conjugated insulating polymer functions as an electron‐capturing mediator to boost interfacial charge transfer and separation, contributing to considerably enhanced photocatalytic selective organic transformation and solar CO_2_ conversion under visible light irradiation.

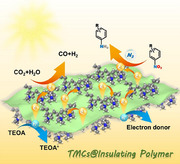

## 1 Introduction

Developing stable, robust, and sustainable artificial photosystems represents an emerging direction to tackle the deteriorating energy crisis.^[^ ^1^ ^]^ Despite the impressive progress, the “Holy Grail” of photocatalysis has still been confined to achieving precise, tunable, and high‐efficiency charge transfer modulation at the micro level, which remains far from satisfactory.^[^ ^2^ ^]^ Among the diverse strategies utilized for reinforcing photoactivity enhancement, co‐catalyst adornment has been widely accepted as a readily accessible, efficacious, and convenient route to boost the interfacial charge separation by directionally withdrawing electrons or holes to the ideal active sites for stimulating photoredox catalysis.^[^ ^3^ ^]^ Although recent years have witnessed explosive investigations on the fabrication of co‐catalyst‐functionalized photocatalytic systems, all these works inevitably suffer from relatively tedious preparative procedures, uneven co‐catalyst dispersion over the whole substrate framework, and inaccessible interface configuration regulation.^[^ ^4^ ^]^ As such, optimizing the integration mode between the co‐catalyst and semiconductor substrate remains challenging for maximally boosting the charge separation efficiency. Moreover, reduction co‐catalysts in the photocatalysis focus on transition metal phosphides, metal sulfides, and noble metal nanoparticles. Up to date, there is a deficiency of novel co‐catalyst alternatives that could drive the efficient charge flow, and simultaneously inherit the merits of conventional co‐catalysts.^[^ ^5^ ^]^

Unlike conventional conjugated polymers, non‐conjugated polymers, which occupy the primary category of polymers, have been rarely utilized as co‐catalysts owing to their generic scarcity of π conjugation in the molecule chain, rendering the charge transport ability of non‐conjugated polymers in principle inaccessible.^[^ ^6^ ^]^ However, our group has verified that non‐conjugated polyelectrolytes (PEs) are actually able to serve as charge transport regulators to boost charge separation over transition metal chalcogenides (TMCs).^[^ ^7^ ^]^ Then, there comes an essential question: what driving force endows the non‐conjugated PEs with unexpected charge transport capability, and what influence do non‐conjugated PEs exert on the photocatalytic mechanism from the perspective of the micro level? The answers to these questions are of paramount significance to unveil the root origins accounting for the unanticipated charge transfer property of non‐conjugated polymers, and more importantly, to substantially push forward the construction of diverse non‐conjugated polymer‐based artificial photosystems for photocatalysis.

Herein, commercially available PE of poly(diallyldimethylammonium chloride) (PDDA) with protonation of amine groups is selected as the non‐conjugated polymer co‐catalyst for photoredox catalysis. The ultrathin PDDA layer is uniformly and seamlessly coated on the TMCs substrates for customizing TMCs@PDDA photocatalytic systems toward multifarious photocatalysis. The results unlock that PDDA does not influence the crystal phase and optical property of TMCs substrates, but rather efficaciously accelerates the interfacial electron flow and boosts charge separation owing to the existence of an electron‐deficient quaternary ammonium functional group in the molecular structure of PDDA. As a result, the self‐assembled TMCs@PDDA nanocomposites demonstrate significantly boosted and versatile photoactivities for photocatalytic aromatic nitro compounds reduction and CO_2_‐to‐syngas conversion under visible light irradiation. The essential roles of PDDA are unambiguously investigated by the integrated experimental and theoretical investigation, including increasing reactant adsorption capacity, providing enriched active sites, and accelerating interfacial carrier transport and separation.

## 2 Results and Discussion

**Figure 1:**
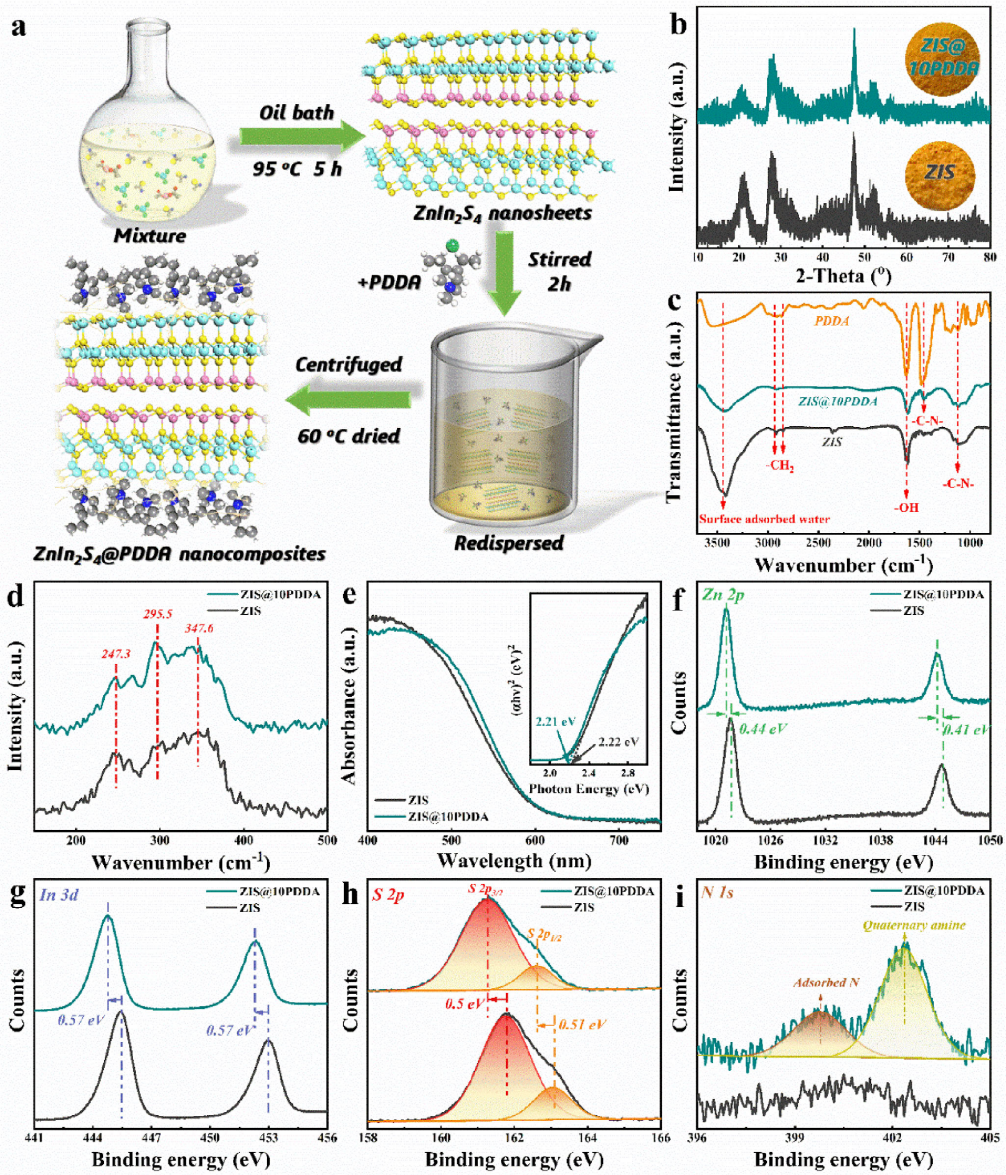
a) Schematic demonstration of the procedures for preparing ZIS@PDDA composite photosystem. b) XRD (inset: photographs), c) FTIR, d) Raman spectra of ZIS and ZIS@10PDDA nanocomposite. e) DRS results of ZIS and ZIS@10PDDA nanocomposite with bandgap determination plots. high‐resolution spectra of ZIS and ZIS@10PDDA nanocomposite including f) Zn 2p, g) In 3d, h) S 2p, and i) N 1s spectra of ZIS and ZIS@10PDDA nanocomposite.

The procedure for the synthesis of ZIS@PDDA nanocomposites is concisely depicted in the schematic diagram (**Figure** **1**a). First, pristine ZnIn_2_S_4_ nanosheets (ZIS NSs) were prepared by a wet‐chemistry method, followed by re‐dispersion in PDDA aqueous solution with different concentrations to form the ZIS@PDDA nanocomposites. As shown in Figure S1 (Supporting Information), ZnIn_2_S_4_ nanosheets demonstrate a negatively charged surface (−23.7 mV), which promotes the electrostatic attraction of positively charged PDDA molecules with quaternary amine functional group.^[^ ^8^ ^]^ After PDDA modification, the zeta potential of ZIS@10PDDA nanocomposite changes to be positive (+12.3 mV), indicating PDDA has been successfully grafted onto the surface of ZIS nanosheets.

According to the XRD results, all the peaks obtained from ZIS and ZIS@10PDDA can be indexed to the hexagonal ZnIn_2_S_4_ (JCPDS card no. 65‐2023) (Figure 1b).^[^ ^9^ ^]^ Notably, PDDA encapsulation cannot be directly reflected by the XRD results because no crystalline phase is observed. As displayed in Figure 1c, FTIR results of the samples are probed, in which the bands at 3450 and 1624 cm^−1^ correspond to the surface adsorbed water and hydroxyl groups.^[^ ^10^ ^]^ The peaks at 1466 and 1130 cm^−1^ are related to the C─N stretching vibration mode from PDDA molecules.^[^ ^11^ ^]^ FTIR spectrum of ZIS@10PDDA shows the C─N peak compared with blank ZIS, verifying PDDA coating on the ZIS surface. These functional groups are beneficial for modifying the ZIS surface, resulting in intimate interaction between PDDA and the ZIS matrix. As for the Raman results of ZIS and ZIS@10PDDA (Figure 1d), three peaks at 247.3, 295.5, and 347.6 cm^−1^ correspond to the longitudinal optical (LO_1_), transverse optical (TO_2_), and LO_2_ modes of ZnIn_2_S_4_, respectively.^[^ ^12^ ^]^ Similarly, no Raman peak attributable to PDDA is observed owing to its intrinsic amorphous property. Figure 1e exhibits the UV–vis diffuse reflectance spectra (DRS) results of ZIS and ZIS@10PDDA nanocomposite. Apparently, PDDA coating on the ZIS surface barely changes the optical feature of ZIS. Consistently, as shown in the inset of Figure 1e, bandgap energies (E_g_) of ZIS (2.22 eV) and ZIS@10PDDA (2.21 eV) calculated from the Kubelka–Munk function are almost the same.^[^ ^13^ ^]^ Hence, PDDA encapsulation on the ZIS surface exerts an ignorable effect on the crystal structure, morphology, and light adsorption of the ZnIn_2_S_4_ matrix. Besides, the specific surface areas of ZIS and ZIS@10PDDA were measured as 19.44 and 1.89 m^2^ g^−1^, respectively (Figure S2 and Table S2, Supporting Information). The decrease in total pore volume from 0.034 cm^3^ g^−1^ (ZIS) to 0.011 cm^3^ g^−1^ (ZIS@10PDDA) and the increase in average pore size from 4.44 nm (ZIS) to 12.87 nm (ZIS@10PDDA) are observed. This significant reduction in specific surface area and porosity after PDDA encapsulation can be attributed to the aggregation of ZIS induced by the PDDA. Nevertheless, the subsequent comparative experiments have verified that the photocatalytic performances of ZIS@10PDDA are not influenced by the reduced specific surface area and porosity but rather the interfacial charge transport/separation mediated by PDDA.

From the survey X‐ray photoelectron spectra (XPS) of ZIS and ZIS@10PDDA (Figure S3a, Supporting Information), Zn 2p, In 3d, S 2p, N 1s, and C 1s peaks are clearly observed, among which N 1s is mainly from the PDDA coating. In the Zn 2p spectrum of ZIS (Figure 1f), the peaks centered at 1021.58 and 1044.56 eV are indexed to Zn^2+^.^[^ ^14^ ^]^ Compared with blank ZIS, binding energies (B.Es) of Zn 2p_1/2_ (1021.14 eV) and Zn 2p_3/2_ (1044.15 eV) are blue shifted ca. 0.41–0.44 eV, implying electronic interaction occurs between ZIS and PDDA. Figure 1g shows the In 3d spectra of ZIS and ZIS@10PDDA, both of which validate the existence of In^3+^.^[^ ^15^ ^]^ S 2p spectrum of ZIS (Figure 1h) comprises a spin–orbit coupling with B.Es. of 161.81 (S 2p_3/2_) and 163.15 eV (S 2p_1/2_), indicative of the existence of S^2−^ species.^[^ ^9^ ^]^ The similar negative shift in B.Es. In the 3d spectrum of ZIS@10PDDA, when compared with pristine ZIS is also observed that there is, In 3d (~0.57 eV) and S 2p (~0.5 eV), which once again suggests a strong interaction between encapsulated PDDA and ZIS substrate. It can be inferred that there is charge transfer between ZIS and PDDA to balance the surface charge distribution, which probably provides a reliable photochemical platform for favorable charge transfer regulation.^[^ ^16^ ^]^ Additionally, as shown in Figure 1i, the N1s spectrum of pristine ZIS does not show an N signal. Conversely, a clear signal is seen in the N1s spectrum of ZIS@10PDDA, which is deconvoluted into 399.71 and 402.40 eV, which correspond to the absorbed N and quaternary amine from the PDDA layer (Figure S4, Supporting Information), respectively.^[^ ^7^, ^17^ ^]^ C 1s spectrum of ZIS@10PDDA nanocomposite (Figure S3b, Supporting Information) shows three peaks at 284.80 (C─C), 286.00 (C─N), 286.69 (C─O) and 287.92 (C═O) eV,^[^ ^7^, ^18^ ^]^ whereas, no C─N peak is detected in the C1s spectrum of ZIS, suggesting again that the nitrogen‐containing functional groups originates from PDDA encapsulation. Together, these results indicate that PDDA was coated onto the ZIS by electrostatic self‐assembly.

**Figure 2:**
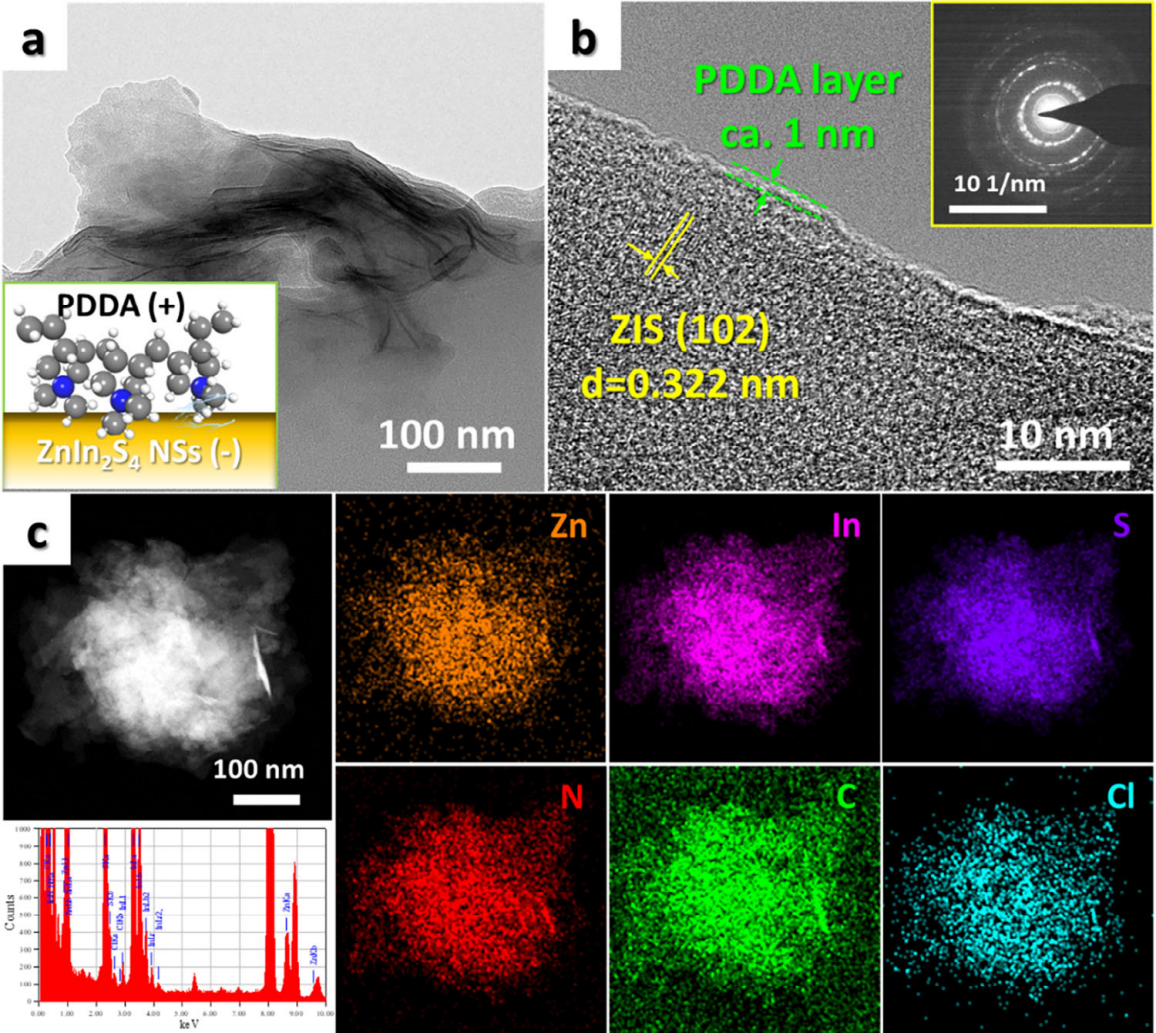
a) TEM and b) HRTEM images of ZIS@10PDDA with SAED result in the inset; c) TEM‐EDX mapping result of ZIS@10PDDA nanocomposite.

The morphologies of the catalysts were characterized by transmission electron microscopy (TEM). The ZIS nanosheets are featured by an ultra‐thin 2D configuration (Figure S5, Supporting Information), and the inter‐planar spacing was measured to be 0.322 nm, which agrees well with the (102) crystal plane of ZnIn_2_S_4_.^[^ ^9^ ^]^ Additional SEM imaging and energy dispersive spectroscopy (EDS) results (Figure S6, Supporting Information) verify the compositional uniformity of the as‐synthesized ZIS substrate, which agrees with the TEM image (**Figure** **2**a). The nanosheet structure can provide a large specific surface area to enrich the active sites and shorten the diffusion distance of charge carriers. After PDDA encapsulation (Figure 2b; Figure S7, Supporting Information), it can be seen that an ultra‐thin amorphous PDDA with a thickness of ≈1 nm is coated on the surface of ZIS. The corresponding HETRM image of ZIS@10PDDA also shows (Figure 2b) the interplanar spacing of 0.322 nm corresponding to the (102) crystal face of hexagonal ZnIn_2_S_4_. Note that the diffraction fringes of ZIS@10PDDA in the selected area electron diffraction (SAED) result demonstrate the lower crystallinity than that of pure ZIS, verifying the amorphous property of the PDDA layer. The distribution of Zn, In, S, N, C, and Cl elements determined from the elemental mapping (Figure 2c) reveals that PDDA is homogeneously coated in the whole framework of ZIS nanosheets.

**Figure 3:**
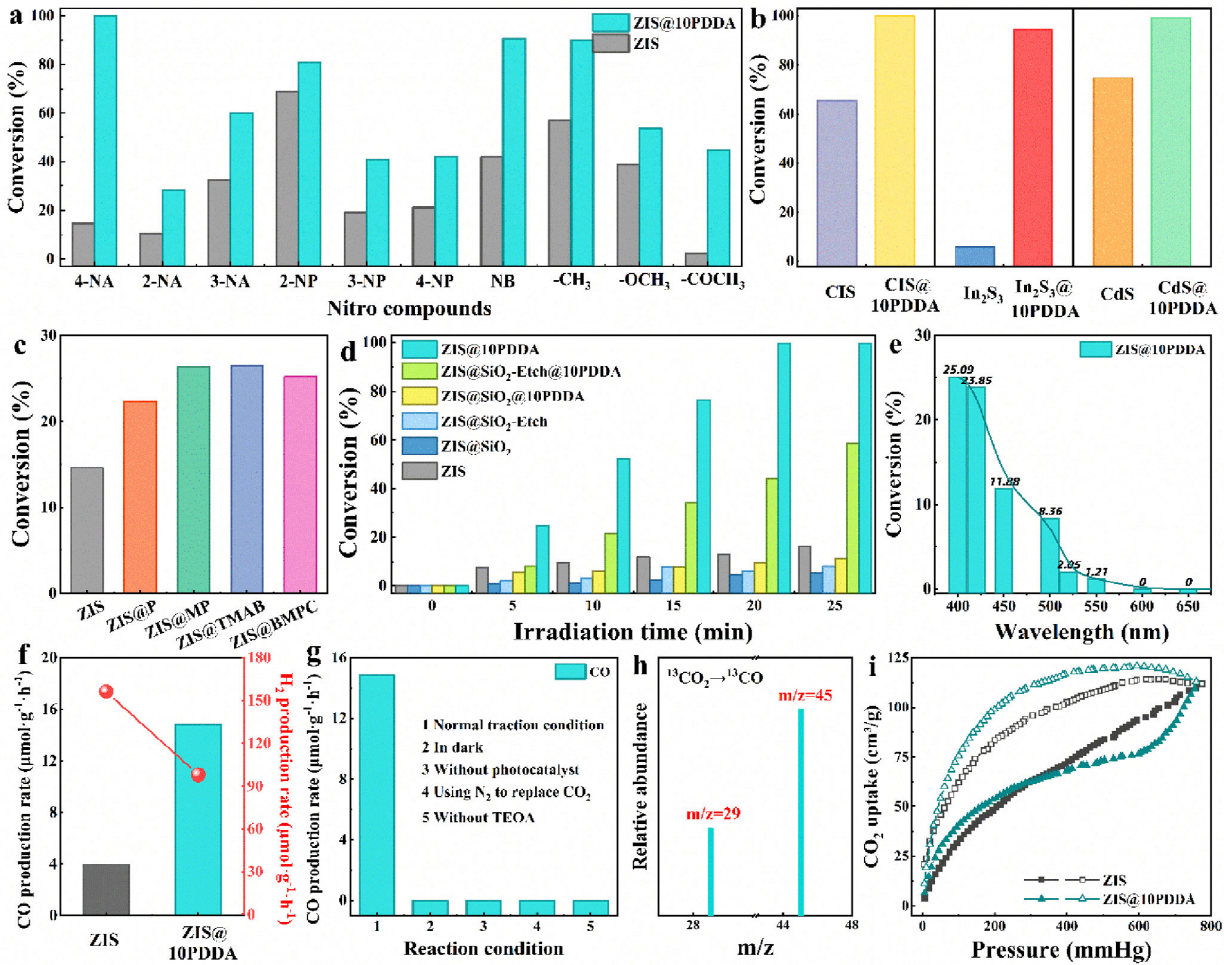
a) Photoactivities of pristine ZIS and ZIS@10PDDA toward selective photoreduction of aromatic nitro compounds, including 4‐NA, 2‐NA, 3‐NA, 2‐NP, 3‐NP, 4‐NP, NB, 4‐nitrotoluene, 4‐nitroanisole and 4‐nitroacetophenone under visible light (λ>420 nm); b) photoactivities of CIS vs. CIS@10PDDA, In_2_S_3_ vs. In_2_S_3_@10PDDA, and CdS vs. CdS@10PDDA toward photoreduction of 4‐NA; c) photoactivities of ZIS and ZIS@amine‐containing organic molecules for 4‐NA photoreduction; d) photoactivities of ZIS, ZIS@SiO_2_, ZIS@SiO_2_‐Etch, ZIS@SiO_2_@10PDDA, ZIS@SiO_2_‐Etch@10PDDA, and ZIS@10PDDA for photoreduction of 4‐NA; e) 4‐NA photoreduction over ZIS@10PDDA under monochromatic light; f) CO_2_ photoreduction activities of ZIS and ZIS@10PDDA; g) CO_2_ photoreduction performances of ZIS@10PDDA under different reaction conditions; h) mass spectrum of ^13^CO (*m/z* = 29) produced over ZIS@10PDDA in photocatalytic reduction of ^13^CO_2_; i) CO_2_ adsorption isotherms of ZIS and ZIS@10PDDA (273 K).

Photocatalytic performance of ZIS@10PDDA nanocomposite was probed by photocatalytic reduction of aromatic nitro compounds and CO_2_ photoreduction under visible light (λ>420 nm) irradiation. Figure S8 (Supporting Information) shows the photocatalytic activities of ZIS@xPDDA with PDDA concentration changing from 5 to 60 mg mL^−1^. Taking a view of the overall photoactivities, it is apparent that all the ZIS@xPDDA nanocomposites exhibit the sharply enhanced photoactivities compared with pristine ZIS, and the optimal photocatalytic performance is obtained when the PDDA concentration is controlled at 10 mg mL^−1^. As displayed in **Figures** **3**a and S9 (Supporting Information), consistently, the optimal ZIS@10PDDA nanocomposite also exhibits the improved photocatalytic performances with respect to pure ZIS for photoreduction of a collection of nitro aromatics with different substituent groups, such as 2‐nitroaniline (2‐NA), 3‐nitroaniline (3‐NA), 2‐nitrophenol (2‐NP), 3‐nitrophenol (3‐NP), 4‐nitrophenol (4‐NP), nitrobenzene (NB), 4‐nitrotoluene, 4‐nitroanisole and 4‐nitroacetophenone. As reflected by Figures S10 and S11 (Supporting Information), pure PDDA shows negligible light absorption and photocatalytic activity, which strongly suggests that photo‐induced charge carriers of ZIS substrate are indispensable to stimulate the photocatalytic organic transformation reactions.

A similar result is observed for a series of TMCs@10PDDA nanocomposites for the photoreduction of aromatic nitro compounds. Specifically, CdIn_2_S_4_ (CIS), In_2_S_3_, and CdS are utilized to replace the ZIS substrate for constructing the TMCs@10PDDA photosystems to investigate the general roles of TMCs and PDDA in mediating the interfacial charge transport efficiency. As shown in Figure 3b; Figure S12 (Supporting Information), CIS@10PDDA, In_2_S_3_@10PDDA, and CdS@10PDDA nanocomposites exhibit the markedly enhanced photocatalytic performances in comparison with the pristine CIS, In_2_S_3_, and CdS counterparts, highlighting the general role of PDDA in triggering the photocatalytic reactions. Detailed characterization of the TMCs@10PDDA nanocomposites in terms of crystalline structure, optical features, morphology, and elemental components is shown in Figures S13–S16 (Supporting Information).

The analogous modification idea was utilized to prepare ZIS@amine‐containing organic molecules [pyrrolidine (P), 1‐methylprrolidine (MP) tetramethylammonium bromide (TMAB), and 1‐butyl‐1‐methylpyrrolidinium chloride (BMPC)] nanocomposites for photoreduction catalysis to evaluate the general roles of amine‐containing organic molecules in mediating the interfacial charge transport efficiency. XRD results of ZIS@P, ZIS@MP, ZIS@TMAB, and ZIS@BMPC are provided in Figure S18 (Supporting Information), in which the peaks correspond to the hexagonal ZnIn_2_S_4_ without the appearance of new peaks, indicating structural integrity of the ZIS substrate. Besides, ZIS@P, ZIS@MP, ZIS@TMAB, and ZIS@BMPC exhibit the analogous light absorption to ZIS with almost an equal bandgap (2.22 eV) (Figure S19, Supporting Information). As displayed in Figure 3c and Figure S17 (Supporting Information), all the ZIS@amine‐containing organic molecules nanocomposites exhibit enhanced photoactivity compared with pure ZIS, implying the important role of tertiary/quaternary amine as electron‐withdrawing mediators to boost the charge separation. Besides, it is clear that ZIS@10PDDA exhibits the most improved photoactivity in comparison with the counterparts of ZIS@amine‐containing organic molecule nanocomposites. This result corroborates that quaternary amine groups from PDDA play the most important role in benefiting the photoreduction catalysis.

Control experiments were then conducted to investigate the photocatalytic mechanism of ZIS@10PDDA composites toward the reduction of 4‐NA. Blank experiments without light or a catalyst (Figure S20a, Supporting Information) show no conversion of 4‐NA, verifying that it is a photocatalytic reaction. Photocatalytic activity of ZIS@10PDDA is considerably decreased once K_2_S_2_O_8,_ as an electron scavenger, was added into the reaction (Figure S20b, Supporting Information), implying the electron‐driven photoreduction reaction. Moreover, Figure S20c,d (Supporting Information) manifests that photocatalytic activities of ZIS, ZIS+10PDDA, and ZIS@10PDDA were considerably decreased when a hole scavenger was not added into the reaction system, verifying that ammonium formate is crucial to boost the charge separation. It should be emphasized that ZIS+10PDDA was prepared by mixing ZIS with PDDA to investigate the role of the ZIS‐PDDA interface in dictating the photoactivity enhancement. It reveals that the photocatalytic activity of ZIS+10PDDA is much lower than that of ZIS@10PDDA, whether the hole scavenger is added or not. This is attributed to the weak interaction between ZIS and PDDA in ZIS+10PDDA, which is not able to induce the efficacious interfacial charge transfer, signifying the importance of interfacial interaction between ZIS and PDDA in boosting the charge transport. However, ZIS+10PDDA still demonstrates more improved photocatalytic activity than ZIS, especially with adding the hole scavenger (NH_4_COOH), which is mainly attributed to the crucial role of PDDA as an electron‐withdrawing mediator. Moreover, calcining ZIS@10PDDA inN_2_wasperformed to additionally investigate the interaction between ZIS and PDDA. As shown in Figure S21 (Supporting Information), ZIS@10PDDA calcined at 200 °C demonstrates considerably reduced photocatalytic activity in comparison with ZIS, and the photoactivity reduces as the calcined temperature increases. This is stemmed from the carbonization of PDDA, and therefore, PDDA gradually decomposes with molecular structure destroyed, which agrees with the TG result (Figure S22, Supporting Information). Characterizations of ZIS@10PDDA calcined at different temperatures are shown in Figures S23 and S24 (Supporting Information), indicating that the samples maintain the structural integrity and optical features of the ZIS. Thus, photoactivity variation of calcined ZIS@10PDDA results from the change of PDDA rather than the ZIS substrate.

The contributing role of PDDA in photocatalytic reaction can also be revealed by the photocatalytic activity of ZIS@10PDDA with a SiO_2_ insulating layer coated at the interface of ZIS and PDDA. As shown in Figure 3d, ZIS@SiO_2_ exhibits considerably reduced photocatalytic performance in comparison with ZIS, which is primarily because of the shielding effect afforded by the insulating SiO_2_ interim layer.^[^ ^19^ ^]^ ZIS@SiO_2_@10PDDA, which was prepared by coating PDDA (10 mg mL^−1^) on the surface of ZIS@SiO_2_, demonstrates enhanced photocatalytic performance than ZIS@SiO_2_ (5.45%). After etching by NaOH (0.2 m), the photoactivity of the thus‐obtained ZIS@SiO_2_‐Etch rebounds to 7.95%. Similarly, ZIS@SiO_2_‐Etch@10PDDA (58.7%) with PDDA coated on the outermost layer of ZIS@SiO_2_‐Etch demonstrates significantly enhanced photoactivity than ZIS@SiO_2_@10PDDA (11.2%). The results concurrently imply that the interim insulating SiO_2_ layer cuts off the interfacial charge transport between ZIS and PDDA, thus blocking the carrier flow from ZIS to PDDA. That is, PDDA indeed acts as an electron‐trapping regulator to boost charge separation. The morphologies of ZIS@SiO_2_, ZIS@SiO_2_‐Etch, and ZIS@SiO_2_‐Etch@10PDDA are characterized by the TEM images and elemental mapping results (Figures S25–S27, Supporting Information).

As shown in Figure 3e, the action spectrum of ZIS@10PDDA toward 4‐NA reduction was probed. Photocatalytic 4‐NA conversion is observed under light with a wavelength varying from 400 to 600 nm, which agrees with the DRS result (Figure 1e), implying the photoexcitation of ZIS contributes to the photoactivity of ZIS@10PDDA. Besides, stability is also an important factor in measuring the application prospect of the catalyst. As shown in Figure S28 (Supporting Information), ZIS@10PDDA can preserve ≈50% of the initial photoactivity after successive 5 runs, which is much better than pristine ZIS. The favorable photostability of ZIS@10PDDA nanocomposite suggests that photo‐corrosion of ZnIn_2_S_4_ can be inhibited by coupling with PDDA. In addition, phase structure, elemental composition, electronic state, and morphology of ZIS@10PDDA retains intact after the cyclic reaction (Figures S29–S31, Supporting Information), confirming the good photostability of ZIS@10PDDA nanocomposite.

Besides the photocatalytic nitro aromatics reduction, the photocatalytic CO_2_ reduction reaction was further conducted to explore the photocatalytic performances of the catalysts. As exhibited in Figure 3f, H_2_ and CO are found to be the major products of the CO_2_ photoreduction reaction. The CO production rate and selectivity increase, which are almost 3.8 and 5.3 times relative to those of blank ZIS, indicating ZIS@10PDDA shows the enhanced CO production rate and selectivity. Figure S32 (Supporting Information) indicates that the CO evolution rate of ZIS@10PDDA increases with elevating the light intensity, indicative of a photocatalytic process. Control experiments were performed in the absence of visible light, photocatalyst, or TEOA or by replacing CO_2_ with N_2_, as shown in Figure 3g. The absence of light irradiation, photocatalyst, or TEOA leads to negligible product detection, indicating the reaction is driven by the photons of the photocatalyst, and the sacrificial agent is indispensable. When CO_2_ was changed to inert N_2_, almost no CO was detected, implying that the products mainly derived from the decomposition of carbon residues on photocatalysts are negligible. As displayed in Figure S33 (Supporting Information), the sacrificial reagent substantially influences the CO evolution rate of ZIS@10PDDA, among which TEOA demonstrates the best efficiency. The action spectrum of ZIS@10PDDA toward CO_2_ photoreduction was also explored. As exhibited in Figure S34 (Supporting Information), efficient CO_2_‐to‐CO reduction is observed under light irradiation from 400 to 600 nm, which agrees with the DRS result and action spectrum of ZIS@10PDDA for 4‐NA photoreduction, suggesting the photoactivity of ZIS@10PDDA results from the photoexcitation of ZIS substrate. Cyclic photocatalytic CO_2_ reaction of ZIS@10PDDA is also carried out (Figure S35, Supporting Information). As displayed in Figure 3h, an isotope experiment was carried out to trace the carbon source of CO. The peaks at *m/z* = 29 confirm that the source of the produced CO is from ^13^CO_2_ gas rather than other organic species in the photocatalytic system. Besides, appreciable CO_2_ adsorption amounts of ZIS and ZIS@10PDDA were probed, as displayed in Figure 3i. Apparently, ZIS@10PDDA shows a slightly higher CO_2_ capture amount than ZIS, which is attributed to the Lewis acid‐base interaction between PDDA and absorbed CO_2_ molecules.^[^ ^20^ ^]^

**Figure 4:**
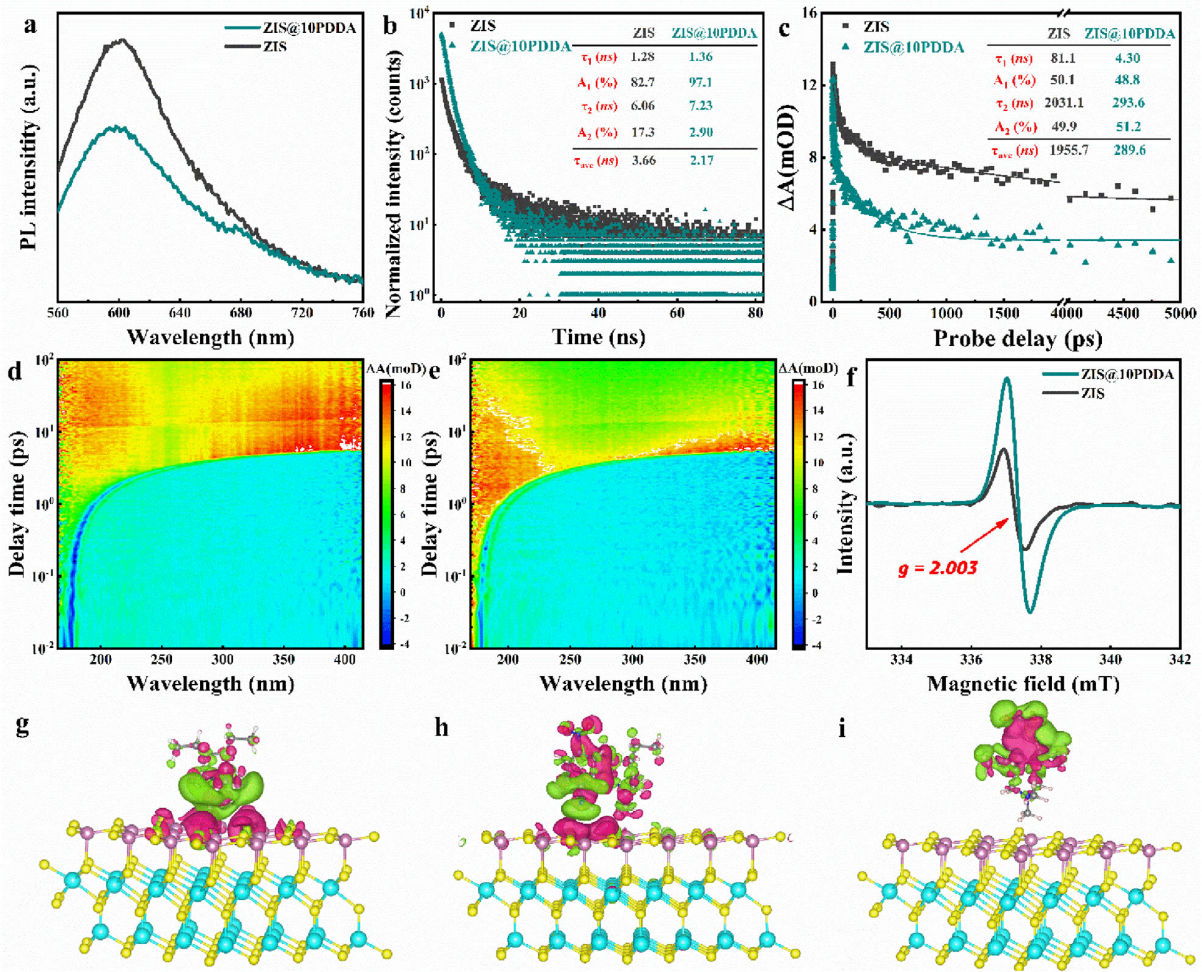
Evaluation of photo‐induced charge separation efficiency. a) PL spectra of ZIS and ZIS@10PDDA nanocomposite. b) Time‐resolved transient PL decay results of ZIS and ZIS@10PDDA nanocomposite (λ_ex_ = 540 nm). c) Ultrafast TA spectra of ZIS and ZIS@10PDDA. The TA signal (i.e., differential absorbance, ΔA) is given in mOD, where OD represents the optical density. 2D plots of TA spectra for d) ZIS and e) ZIS@10PDDA photoexcited at 400 nm, wherein the horizontal axis, vertical axis, and color scale represent the probe wavelength, pump‐probe time delay, and TA signal, respectively. f) EPR results of ZIS and ZIS@10PDDA. The result of charge density difference (green: charge depletion, violet: charge accumulation) for the most energy favorable model of g) PDDA absorbed on the ZIS, and h) 4‐NA and i) CO_2_ molecules absorbed on the ZIS@PDDA.

Since photoluminescence (PL) originates from the charge recombination, photochemical charge separation can thus be analyzed by PL emission spectroscopy. As shown in **Figure** **4**a, PL spectra of ZIS show a substantial peak at 600 nm, which corresponds to the radiative self‐trapped excitation recombination. After PDDA encapsulation, PL emission intensity of ZIS markedly decreases, which indicates that ZIS@PDDA exhibits a slower charge recombination process with a longer decay lifetime. Migration dynamics of charge carriers were investigated by time‐resolved transient photoluminescence (TRPL) decay spectra (Figure 4b). The curves are fitted by the bi‐exponential decay kinetic equation below^[^ ^13^ ^]^:

(1) $$\tau_{\mathrm{ave}\;}=\left(A_{1}{\tau_{1}}^{2}+A_{2}{\tau_{2}}^{2}\right)/\left(A_{1}\tau_{1}+A_{2}\tau_{2}\right)$$

where A_1_ and A_2_ represent the fractional contributions from time‐resolved emission decay lifetime τ_1_and τ_2_. The average luminous lifetime of ZIS@10PDDA (2.17 ns) is obviously faster than ZIS (3.66 ns), which is because the PDDA coated on the ZIS surface can trigger a preferential electron transfer channel, thereby facilitating the spatial charge separation.

To comprehend the improved charge separation efficiency, ultrafast pump‐probe transient absorption (TA) spectroscopy was applied to study the carrier transport dynamics of ZIS@10PDDA nanocomposite. As shown in Figure 4c, pristine ZIS shows a stimulated emission (SE) signal, which is featured by two time constants: τ_1_ = 81.1 ns (50.1%) and τ_2_ = 2031.1 ns (49.9%), and the average lifetime is determined as 1955.7 ns. Similar TA profiles are observed for ZIS@10PDDA, which can also be characterized by two time constants: τ_1_ = 4.3 ns (48.8%) and τ_2_ = 293.6 ns (51.2%), and the weighted average lifetime is 289.6 ns. Obviously, ZIS@10PDDA nanocomposite exhibits a nearly seven‐fold acceleration of free charge carrier relaxation with respect to blank ZIS (Figure 4d,e), suggesting the rapid electron migration from the ZIS to PDDA. Thereby, the above results verify that PDDA decorated ZnIn_2_S_4_ nanohybrids can significantly facilitate the spatial charge separation and provide more opportunities for electrons to be involved in the subsequent photoreduction catalysis.

To further prove the critical role of PDDA in fostering the photoelectron transfer and charge separation, photoelectrochemical (PEC) measurements were carried out under visible light irradiation. As shown in Figure S36a (Supporting Information), ZIS@10PDDA nanocomposite exhibits a smaller arc radius than ZIS, suggesting its lower interfacial charge transfer resistance than ZIS. Additionally, the fitted electrochemical impedance spectroscopy (EIS) results of the photoelectrodes are provided in the inset. As unveiled by the fitted results (Table S4, Supporting Information), ZIS@10PDDA nanocomposite (5434 Ω) indeed exhibits the lower charge transport resistance (*R_ct_*) relative to ZIS (6514 Ω) under visible light irradiation, indicating its more efficient interfacial charge transport efficiency. The open‐circuit potential results (Figure S36b, Supporting Information) show that ZIS@10PDDA nanocomposite exhibits the larger photovoltage in comparison with ZIS, confirming its more efficient charge separation. It is worth noting that ZIS@10PDDA nanocomposite shows a longer electron lifetime than ZIS (Figure S36b, Supporting Information, inset), which agrees with the EIS results and photocatalytic performances. Cyclic Voltammetry (CV) curves are applied to study the possible redox potential of PDDA (Figure S37, Supporting Information). It is obvious that there is no difference among the CV curves of carbon paper and PDDA with different concentrations, which substantiates that PDDA is indeed a non‐conjugated polymer without HOMO‐LUMO energy band structure.

Besides, formation of V_Zn_ was confirmed by the observation of apparently symmetric peaks at g ≈ 2.003 in the Electron paramagnetic resonance (EPR) result (Figure 4f).^[^ ^21^ ^]^ Notably, ZIS@10PDDA nanocomposite exhibits a more enhanced peak intensity than pristine ZIS, indicating its higher V_Zn_ concentration. The broadening in ΔH corresponds to the imbalanced charge and local electric field around the V_Zn_, which gives rise to the suppression of the lattice relaxation phenomenon and shortens the lattice relaxation time (ΔH ∝ 1/T).^[^ ^22^ ^]^ Thus, the presence of abundant V_Zn_ in ZIS after PDDA encapsulation promotes electron capturing and inhibits photo‐corrosion through the suppressed lattice relaxation, resulting in the enhanced charge separation. Meanwhile, to further explore the interaction between PDDA and ZIS, adsorption energies (E_ad_) of different absorption configurations of PDDA on the ZIS surface are compared by Density Functional Theory (DFT) calculations. According to Equation S1 (Supporting Information), the negative adsorption energy leads to the configuration with higher stability.^[^ ^23^ ^]^ As shown in Figure S38 (Supporting Information), we studied the different adsorption positions at which the PDDA moves to the ZIS to adopt the most stable absorption configuration. It can be seen that the most stable absorption configuration is type IV (E_ad_ = −4.790 eV). The corresponding visual representation of charge transfer upon PDDA adsorption on ZnIn_2_S_4_ (001) is shown in the isostructural charge density difference plots (Figure 4g). There are significant charge shifts from PDDA to electronegative ZIS (0.62 e) to form a stable configuration, implying the robust charge accumulation capability of ZIS.^[^ ^24^ ^]^ Subsequently, the type‐IV configuration was utilized to calculate the absorption energies of 4‐NA and CO_2_. Regarding 4‐NA adsorption on the ZIS monolayer (Figure 4h), the lowest adsorption energy value is obtained on Zn top (−0.292 eV). For ZIS@10PDDA nanocomposite, E_ad_ values of 4‐NA on top of Zn, middle of Zn‐S, top of S, and PDDA are 0.205, −0.320, −0.168, and −0.093 eV, respectively, which indicates that adsorption of 4‐NA in the middle of Zn‐S takes precedence over other positions. The results indicate that adsorption energies are enhanced by PDDA coating. Subsequently, we also investigated the adsorption energy variation for CO_2_ adsorbed on the ZIS and ZIS@PDDA nanocomposite. The charge density distributions of the most stable CO_2_ adsorption configuration of ZIS are demonstrated in Figure S39 (Supporting Information) with an adsorption energy of −0.009 eV. Compared with ZIS, the top of PDDA is believed to be more favorable than other positions as an electron transfer mediator and active sites for CO_2_ conversion, confirming the higher CO_2_ adsorption amount for the ZIS@10PDDA nanocomposite (Table S5, Supporting Information). The charge density difference plots for CO_2_ adsorbed ZIS and ZIS@PDDA nanocomposite are displayed in Figure S40 (Supporting Information) and Figure 4i. The electron density plots for CO_2_‐adsorbed ZIS@PDDA nanocomposite show no electron density between ZIS and CO_2_. Thus, PDDA plays essential roles in the adsorption or activation of 4‐NA and CO_2_, which are beneficial for improving the photocatalytic performances.

**Figure 5:**
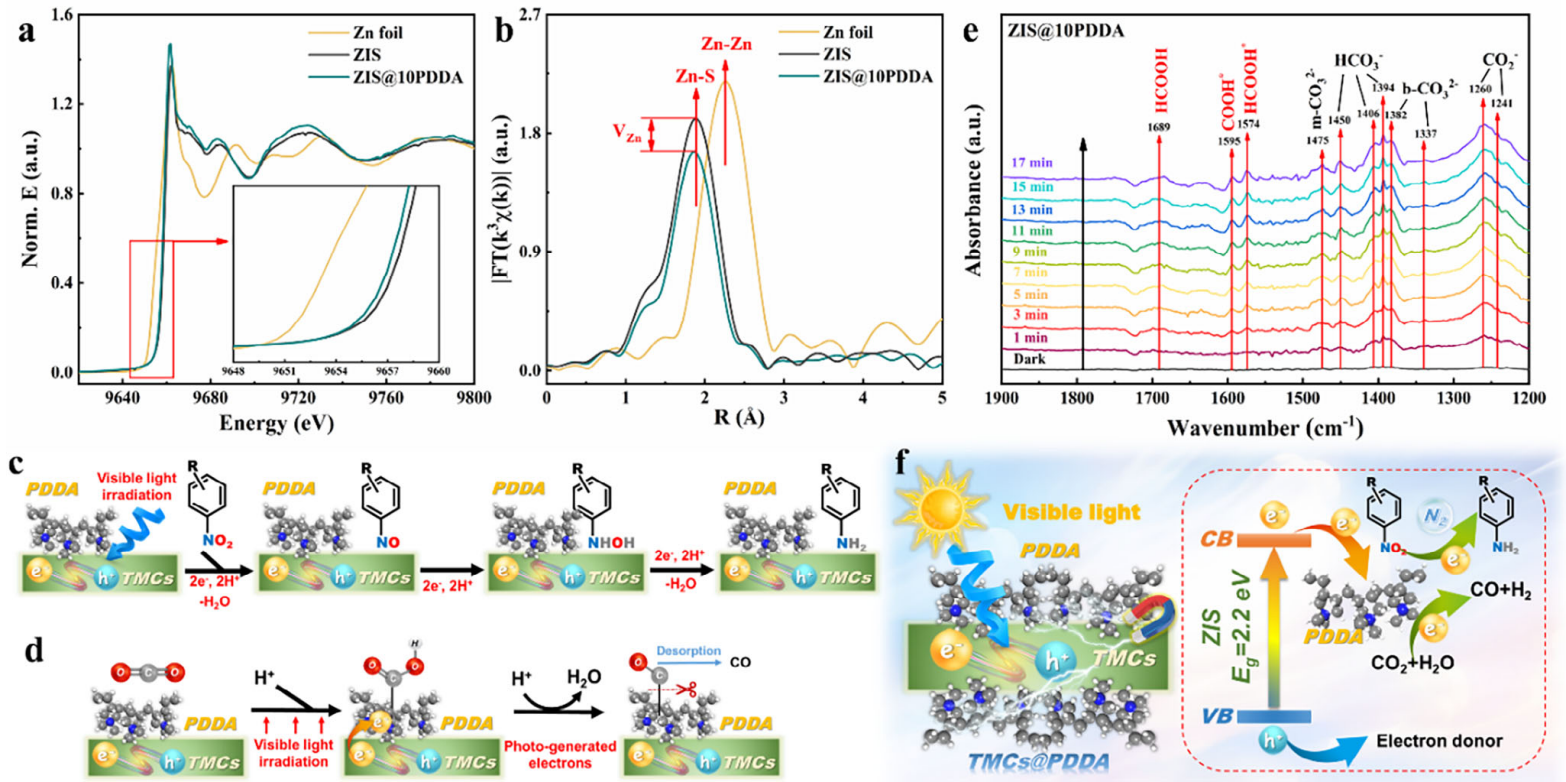
a) Zn K‐edge XANES results of Zn foil, ZIS, and ZIS@10PDDA, b) *k* ^3^‐weighted Fourier transform (FT) χ(*k*) function of the EXAFS results for Zn foil, ZIS, and ZIS@10PDDA nanocomposite. Proposed reaction mechanism for photoreduction of c) 4‐NA and d) CO_2_ over ZIS@PDDA nanocomposite; e) In situ DRIFTS spectra of the CO_2_ photoreduction over ZIS@10PDDA; f) Schematic diagram illustrating the photoredox mechanism of ZIS@PDDA nanocomposite.

To establish a deeper insight into the interfacial interaction of ZIS@10PDDA nanocomposite, the local atomic environment of the Zn atom was analyzed by the X‐ray absorption near‐edge structure (XANES) and extended X‐ray absorption fine structure (EXAFS) results of ZIS before and after PDDA encapsulation. As displayed in the XANES results (**Figure** **5**a), features of the Zn K‐edge in ZIS are similar to those of ZIS@10PDDA, verifying the valence state of Zn^2+^. The absorption edge for ZIS@10PDDA nanocomposite is shifted to lower energy as compared with blank ZIS, which implies partial surface reduction stemming from the unsaturated coordination on the Zn site. Figure 5b shows the Fourier‐transformed EXAFS curves at Zn K‐edge, revealing an intense peak located at around 1.8 Å, corresponding to the Zn─S bond. With the introduction of PDDA, ZIS@10PDDA nanocomposite displays a reduction in the intensity of the Zn─S bond, indicating the emergence of Zn vacancies. This result aligns well with the EPR results.

According to the previous works, the probable pathway for photocatalytic nitro aromatic reduction is shown in Figure 5c. The generation of nitro compounds stems from the gradual generation of nitrosobenzene (Ph‐NO), phenylhydroxylamine (Ph‐NHOH), and eventually aniline (Ph‐NH_2_), following three continuous hydrogenation steps.^[^ ^25^ ^]^ The possible CO_2_ reduction pathway on the surface of ZIS@PDDA is shown in Figure 5d. The adsorbed CO_2_ can be reduced to bent CO_2_^•−^ species, and then a hydrogen atom attacks one oxygen atom in CO_2_^•−^ to yield the carboxyl ^•^COOH, which preferentially integrates with hydrogen radicals to produce the formic acid in water. An immediate bond cleavage between the oxygen atom and the carbon atom is stimulated with a CO molecule left adsorbed, followed by the subsequent desorption of the newly formed carbon monoxide molecule from the catalyst surface.^[^ ^26^ ^]^ In situ DRIFTS analysis of ZIS@10PDDA nanocomposite was provided to confirm the CO_2_ reduction process, as depicted in Figure 5e. It can be seen that a collection of infrared (IR) peaks corresponding to CO_2_^−^ (1241, 1260 cm^−1^), bidentate carbonate species (b‐CO_3_^2−^, 1337, 1382 cm^−1^), bicarbonates (HCO_3_^−^, 1394, 1406, 1450 cm^−1^), and monodentate carbonates (m‐CO_3_^2−^, 1475 cm^−1^) are observed and strengthened with prolonging the reaction time.^[^ ^27^ ^]^ In particular, the crucial intermediates, including HCOOH^*^ (1574 cm^−1^), COOH^*^ (1595 cm^−1^), and HCOOH (1689 cm^−1^), are probed during the photocatalytic CO_2_ reduction reaction.^[^ ^27^, ^28^ ^]^ Hence, CO_2_ photoreduction into CO in our case follows the carbene generation pathway using a single‐metal‐site combined mode (Zn─C or Zn─O bond).

According to the photocatalytic performances, characterization analysis, and DFT calculation, charge transfer characteristics and photoredox mechanism of ZIS@PDDA nanocomposites were proposed (Figure 5e). As displayed in Figure S41 (Supporting Information), ZIS and ZIS@10PDDA show the CB potentials at ≈−0.87 and −0.95 V vs NHE, respectively. Combined with their band gaps, VB potentials of ZIS and ZIS@10PDDA are calculated to be 1.35 and 1.26 V vs NHE based on the formula: E_VB_ = E_CB_ + E_g_. When ZIS is band‐gap‐excited to produce electron‐hole charge carriers under visible light, the PDDA as an electron‐withdrawing mediator and active site accelerates the interfacial charge transfer and fosters the adsorption of reactants, accompanied by a fast release of holes from ZIS to be quenched by sacrificial regent, and simultaneously the photoelectrons take participate in the photocatalytic reduction of aromatic nitro compounds and CO_2_ photoreduction. Therefore, the synergy of electron extraction and reactant adsorption endowed by PDDA guarantees the efficient interfacial charge transfer between PDDA and TMCs, thus facilitating the spatial charge separation and resulting in the excellent photoactivities.

## 3 Conclusion

In summary, we presented the rational design of TMCs@PDDA artificial photosystems and comprehensively unleashed the root reasons accounting for the unexpected charge transport property of non‐conjugated polymer for photoreduction catalysis. It was unambiguously unleashed that PDDA encapsulation on the TMCs substrate benefits the reactant adsorption, and simultaneously electron‐withdrawing capability of PDDA boosts the directional electron transport from TMCs to PDDA, leading to the significantly prolonged charge lifespan. Consequently, the self‐assembled TMCs@PDDA photosystems show remarkably enhanced photocatalytic activities toward the photoreduction of aromatic nitro compounds and CO_2_ photoreduction under visible light irradiation. The photocatalytic mechanism caused by PDDA encapsulation in TMCs@PDDA photosystems was elucidated. Our work would shed light on the smart design of non‐conjugated polymer photosystems and push forward the utilization of non‐conjugated polymer as co‐catalysts for solar energy conversion.

## 4 Experimental Section

The information on the preparation of catalysts, characterization, photocatalytic performances, and photoelectrochemical measurements is specifically provided in the Supporting Information.

## Conflict of Interest

The authors declare no conflict of interest.

## Supporting information



Supporting Information

## Data Availability

The data that support the findings of this study are available from the corresponding author upon reasonable request.
