# Supplementary material for: Maneuvering Charge Transport via Insulating Polymer Interface for Steering Photoredox Catalysis
Source: Adv Sci (Weinh). 2025 Jul 28;12(40):e07670. doi: 10.1002/advs.202507670 (PMC12561469; doi:10.1002/advs.202507670)
Supplement: Supplementary file 1 — Supporting Information [file ADVS-12-e07670-s001.doc]

Supporting Information

**Maneuvering Charge Transport via Insulating Polymer Interface for Steering Photoredox Catalysis**

*Qiao-Ling Mo^a, b^,* *Rui Xiong^b^,* *Bo-Yuan Ning^b^, Peng Su ^b^,* *Qing Chen^b^, Jun-Hao Dong^b^, Bai-Sheng Sa^b^, Jing-ying Zheng^b^, Yue Wu^b^, Fang-Xing Xiao^b, c^**

1. Center of Analysis and Testing, Nanchang University, 999 Xuefu Avenue, Nanchang, Jiangxi Province, 330031, China.
2. College of Materials Science and Engineering, Fuzhou University, New Campus, Minhou, Fujian Province, 350108, China.
3. State Key Laboratory of Structural Chemistry, Fujian Institute of Research on the Structure of Matter, Chinese Academy of Sciences, Fuzhou, Fujian 350002, PR China.

E-mail: fxxiao@fzu.edu.cn

**Experimental Section**

**Chemicals****:** The following chemicals were used as received without further purification: All chemicals were used without further purification. Deionized water (DI H_2_O, Millipore, 18.2 MΩ cm resistivity) was used for all experiments. Zinc acetate dehydrate [Zn(CH_3_COO)_2_·2H_2_O], thioacetamide (TAA), ammonium formate NH_4_HCO_2_), cadmium acetate dihydrate Cd(CH_3_COO)_2_·2H_2_O], potassium persulfate K_2_S_2_O_8_), cadmium chloride hemi(penhydrate) (CdCl_2_·2.5H_2_O), S powder, acetonitrile (C_2_H_3_N), triethanolamine (C_6_H_15_NO_3_, TEOA), methanol (CH_4_O, MeOH), ethanol(C_2_H_6_O, EtOH), ethylene glycol (C_2_H_6_O_2_, MEG), lactic acid (C_3_H_6_O_3_), sodium hydroxide (NaOH), ammonia, dichloromethane (CH_2_Cl_2_) and sodium sulfate (Na_2_SO_4_) were purchased from Sinopharm Chemical Reagent Co., Ltd. Poly(diallyl-dimethyl-ammonium chloride) solution (PDDA, average Mw 200.000-350.000) was obtained from Sigma-Aldrich. Indium chloride (InCl_3_), tetraethyl orthosilicate (TEOS), pyrrolidine (P), 1-methylprrolidine (MP) tetramethylammonium bromide (TMAB), 1-butyl-1-methylpyrrolidinium chloride (BMPC), tetrabutylammonium tetrafluoroborate and relevant aromatic nitro compounds including 2-nitroaniline (2-NA), 3-nitroaniline (3-NA), 4-nitroaniline (4-NA), 2-nitrophenol (2-NP), 3-nitrophenol (3-NP), 4-nitrophenol (4-NP), nitrobenzene (NB), 4-nitrotoluene, 4-nitroanisole, 4-nitroacetophenone were obtained from Aladdin. Deionized water (DI H_2_O, Millipore, 18.2 MΩ cm resistivity) was used for all experiments. All chemicals were of analytical grade and used without further purification.

**Synthesis of photocatalysts:**

*Preparation of ZnIn_2_S_4_ nanosheets (ZIS NSs)*: 300 mL of an aqueous solution containing 395.1 mg of Zn(CH_3_COO)_2_·2H_2_O, 796.3 mg of InCl_3_, and 721.2 mg of TAA was thoroughly stirred for 30 min in a round-bottomed flask, which was then placed at 95 ^o^C and remained for 5 h under vigorous stirring. After cooling down to room temperature, yellow ZnIn_2_S_4_ (ZIS) precipitate was collected by centrifuge and washed with DI H_2_O for three times.

*Preparation of CdIn_2_S_4_ nanosheets (CIS NSs)*: Cd(CH_3_COO)_2_·2H_2_O, InCl_3_ and TAA with the same molar ratio to ZnIn_2_S_4_ were added into a round-bottomed flask, stirred for 30 min and then heated at 100 ^o^C and remained for 12 h under vigorous stirring. After cooling down to room temperature, CdIn_2_S_4_ (CIS) precipitate was collected by centrifuge and washed with DI H_2_O for three times.

*Preparation of In_2_S_3_ nanosheets*: In_2_S_3_ nanosheets were prepared by the same method as utilized for fabricating ZnIn_2_S_4_ without adding Zn(CH_3_COO)_2_·2H_2_O.

*Preparation of CdS nanosheets*: CdS nanosheets were prepared via a hydrothermal method reported previously.^[1]^ In detail, 0.32 mmol CdCl_2_·2.5H_2_O, 2.0 mmol S powder and 12 mL of diethylenetriamine (DETA) were mixed and vigorously stirred to form a homogeneous suspension. Then, the mixture was transfer into a Teflon-lined stainless-steel autoclave with a capacity of 50 mL for 48 h at 80 °C. After cooling to room temperature, a yellowish precipitate was rinsed with deionized water and ethanol separately and dried in an oven at 60 °C for 24 h to obtain the CdS nanosheets.

*Preparation of PDDA decorated TMCs nanosheets*: The Poly(diallyl-dimethyl-ammonium chloride) (PDDA) functionalized TMCs were prepared by a facile method at ambient conditions. The 200 mg of pristine TMCs were suspended in 200 mL of PDDA aqueous solution (5, 10, 15, 30, 60 mg/mL) and vigorously stirred for 2 h. After that, the precipitates (denoted as TMCs@xPDDA, x=5, 10, 15, 30, 60) were centrifuged and dried in vacuum oven at 60 °C for 24 h.

*Preparation of ammonium salt decorated TMCs nanosheets*: The ammonium salt [including in pyrrolidine (P), 1-methylprrolidine (MP) tetramethylammonium bromide (TMAB) and 1-butyl-1-methylpyrrolidinium chloride (BMPC)] functionalized TMCs were prepared by a facile method at ambient conditions. The 200 mg of pristine TMCs were suspended in 200 mL of ammonium salt aqueous solution 10 mg/mL) and vigorously stirred for 2 h. After that, the precipitates (denoted as ZIS@P, ZIS@MP, ZIS@TMAB, and ZIS@BMPC) were centrifuged and dried in vacuum oven at 60 °C for 24 h.

*Preparation of ZIS@SiO_2_ nanocomposites*: The as-prepared ZIS nanosheets (100 mg) was ultrasonically treated in 50 mL of water for 30 min to obtain a well-dispersed ZIS suspension. Under continuous mechanical stirring, 150 mL ethanol and 5 mL of ammonia solution were mixed. 1 mL tetraethyl orthosilicate (TEOS) was then added slowly into the solution and retained at room temperature for 20 min. After the reaction, ZIS@SiO_2_ nanocomposites were filtered and dried under vacuum at 60 ^◦^C for further use.

*Preparation of ZIS@SiO_2_-Etch nanocomposites*: The previously prepared 100 mg ZIS@SiO_2_ nanocomposites dispersed in 100 mL of 0.2 M NaOH aqueous solution. The resulting solution was stirred at the ambient temperature for 2 h. Then, ZIS@SiO_2_-Etch product was collected by centrifugation and washed with water and ethanol several times.

**Photocatalytic selective reduction performances:** For anaerobic photocatalytic reduction of aromatic nitro compounds, a 300 W Xe lamp (PLS-SXE300D, Beijing Perfect Light co. LTD, China) equipped with a 420 nm cut-off filter was used as the light source and kept 15 cm away from the glass reactor. Specifically, 10 mg of catalyst and 40 mg of hole scavenger (NH_4_HCO_2_) were dispersed in 30 mL of aromatic nitro compounds (10 mg/L) under N_2_ bubbling. After keeping in dark for 30 min to achieve the adsorption-desorption equilibrium, the suspension was irradiated with visible light (λ>420 nm). Then, 3 mL of the sample solution was collected at different time (0, 5, 10, 15, 20, and 25 min) and the supernatant was analyzed by UV- vis spectrophotometer (Thermal Fisher, GENESYS, 10S). Photoreduction of other aromatic nitro compounds was carried out under the same conditions.

**Photocatalytic CO_2_ reduction performances:** The photocatalytic CO_2_ reduction was performed in a 25 mL glass reactor. In a typical test run, 10 mg of catalyst was dissolved in an aqueous solution (10 mL) containing 5 mL acetonitrile (MeCN), 2 mL DI H_2_O as a reaction solvent and 3 mL of triethanolamine (TEOA) as the sacrificial agent. The solution was constantly stirred with the help of a magnetic stirrer, preventing settling of particles at the bottom of the reactor. Prior to irradiation, the reaction mixture was vacuumed w for eliminating the dissolved gases, and then the photocatalytic reactor was filled with CO_2_ for 10 mins. The photocatalytic system was thoroughly degassed and then irradiated by a 300W Xe lamp (PLS-SXE300D, Beijing Perfect Light Co. Ltd, China) equipped with a 420 nm-cut-off optical filter (λ>420 nm). A magnetic stirrer was continuously stirred at the bottom of the reactor to keep the catalyst in a suspension status during the whole experiment. The evolving CO and H_2_ were monitored periodically by an online gas chromatograph (Shimadzu GC-2014C using argon as carrier gas). Photoactivities were evaluated based on the CO and H_2_ evolution amount in the first 2 h of the reaction. In the isotope-labeling experiment, ^12^CO_2_ was replaced by ^13^CO_2_. The formation of ^13^CO under identical reaction conditions was analyzed by a Hiden HPR-20 mass spectrometer.

**Characterization**: Morphologies of the samples were probed using a Carl Zeiss Supra55 field-emission scanning electron microscope (FESEM) and Tecnai G2 F20 transmission electron microscope (TEM). The crystal structure was determined by powder X-ray diffraction (XRD) on a Miniflex 600 operated using Cu Ka as the radiation source at 40 kV and 15 mA. The Raman spectroscopy experiments were performed with a DXR2Xi Raman Imaging Microscope from Thermo Fisher Scientific U.S.A. UV-vis diffuse reflectance spectra (DRS) were probed by a Varian Cary 500 using BaSO_4_ as the reference background. X-Ray photoelectron spectroscopy (XPS) measurements were performed on Escalab 250 and binding energy of the elements was calibrated by 284.8 eV. Electron paramagnetic resonance (EPR) spectra were obtained over Bruker A300-10/12 electron paramagnetic resonance spectrometer at room temperature. Photoluminescence spectra (PL) for solid samples were detected on Edinburgh FS5 photoluminescence spectrophotometer and the excitation wavelength was set as 540 nm. Time-resolved photoluminescence (TRPL) spectra were recorded on a FS5 fluorescence lifetime spectrophotometer (Edinburgh Instruments, UK). Fourier transform infrared (FTIR) spectra were recorded on a TJ270-30A infrared spectrophotometer. Zeta potential (ξ) measurements were performed by dynamic light scattering analysis (ZetasizerNano ZS-90). Brunauer-Emmett-Teller (BET) specific surface area, CO_2_ and N_2_ adsorption experiments were carried out on a 3Flex. For the femtosecond TA measurement, we spin-coated (1000 rpm) the prepared samples from the suspensions onto sapphire substrates. The pump-probe techniques were employed with the homebuilt equipment. The samples were pumped at a wavelength of 400 nm in the picosecond TA experiments using a diode-pump Yb medium femtosecond laser system as the excitation source, whose pulse duration, center wavelength and repetition rate were ~190 fs, 1030 nm and 100 kHz, respectively. An optical parametric amplifier (OPA) was also equipped to tune the varied wavelengths from 315 nm to 2600 nm. The white light generated by focusing the laser power into a sapphire crystal was utilized as a probe pulse for the femtosecond TA measurements. In situ DRIFTS measurements were carried out on a Nicolet iS50FT-IR spectrometer (Thermo Fisher, U.S.A.) instrument. The extended X-ray absorption fine structure (EXAFS) and X-ray absorption near-edge spectra (XANES) for Zn K-edge were carried out at the SPring-8. The XAS data were processed using the Athena and Artemis software package.

**Photoelectrochemical (PEC) measurements:** PEC measurements were carried out on electrochemical workstations (CHI 660E and Gamary Interface 1000 E) in a conventional three-electrode quartz cell, which uses a Pt plate as counter electrode, Ag/AgCl electrode as the reference electrode, and the samples coated on FTO were utilized as the working electrodes. The working electrodes were prepared on fluorine-dope tin oxide (FTO) glass that was cleaned by sonication in ethanol for 30 min and dried at 353 K. The boundary of FTO glass was protected using scotch tape. The 5 mg sample was completely dispersed in 0.5 mL of ethyl alcohol absolute by sonication to get slurry which uniformly was spread onto the pretreated FTO glass. After drying in the air, the working electrode was further dried at 80 ^o^C for 2 h to improve adhesion. Then the Scotch tape was unstuck, and the uncoated part of the electrode was isolated with nail polish. The exposed area of the working electrode was 1 cm^2^. Besides, Na_2_SO_4_ (0.5 M, pH=6.69) aqueous solution was used as the electrolyte. Cyclic voltammetry (CV) was probed using a three-electrode system with carbon paper, Pt plate and Ag/AgCl as the working, counter, and reference electrode, respectively. CV was measured in CH_2_Cl_2_ with 0.1 M tetrabutylammonium tetrafluoroborate solution.[[^2^](#_ENREF_1)]

**Computational details:** All DFT calculations were performed by the Vienna ab initio simulation package (VASP)[[^3^](#_ENREF_2)] and dealt with the ALKEMIE platform.[[^4^](#_ENREF_3)] The projector augmented wave (PAW)[[^5^](#_ENREF_4)] was carried out to describe the exchange-correlation functional, while the generalized gradient approximation (GGA)[[^6^](#_ENREF_5)] within Perdew-Burke-Ernzerhof (PBE)[[^7^](#_ENREF_6)] functionals were used to treat the electron-ion interaction. A plane wave basis set with a cut-off energy of 500 eV, and a Gamma k-point mesh were used. The convergence criteria for energy and force were set to be 10^-5^ eV and 0.03 eV/Å. To conform the experimental samples, surface model of ZIS with 7 atomic layers was built from the [001] direction of the bulk ZIS. The 4×4×1 supercell of the ZIS surface was used for the absorption models. A vacuum thickness of 25 Å along the z-axis was added to avoid the adjacent layer interaction. For all the absorption models, the DFT-D3 approach was introduced due to its good description in long-range vdW interactions.[[^8^](#_ENREF_7)] The absorption energies of 4-NA or CO_2_ were calculated by:

$E_{\text{abs}}=E_{\text{ad+sub}}-E_{\text{sub}}-E_{\text{ad}}$ *(S1)*

where *E_ad+sub_* is the total energy of the substrate with absorption molecule, the *E_sub_* is the substrate and the *E_ad_* is the total energy of an isolate 4-NA or CO_2_ molecule.

To further understand the charge transfer from the absorbed molecule to the substrate, the charge density differences $\rho_{\text{diff}}$ were obtained by:

$\rho_{\text{diff}}=\rho_{\text{mole+sub}}-\rho_{\text{mole}}-\rho_{\text{sub}}$ *(S2)*

where $\rho_{\text{mole+sub}}$, $\rho_{\text{mole}}$ and $\rho_{\text{sub}}$ present the charge density of the substrate with absorption molecule, the absorption 4-NA or CO_2_ molecule and substrate, respectively.





**Figure S1.** Zeta potentials of ZIS and ZIS@10PDDA suspension.





**Figure S2.** N_2_ adsorption-desorption isotherms of (a) ZIS and (b) ZIS@10PDDA. Insert: pore size distribution.





**Figure S3.** (a) Survey and (b) high-resolution C1s spectra of ZIS and ZIS@10PDDA.





**Figure S4.** High-resolution (a) N1s and (b) C1s spectra of pure PDDA.

**Note:** High-resolution C 1s spectrum of PDDA (**Figure S4a**) can be deconvoluted into two peaks, in which the peak at 284.80 is assignable to the sp^2^-hybridized carbon (C−C), and the peak at 286.06 eV corresponds to the C-N bond from PDDA. **Figure S4b** shows the high-resolution N1s spectrum of PDDA, in which the central peak at ca. 402.25 eV is attributed to the charged nitrogen N^+^ species.


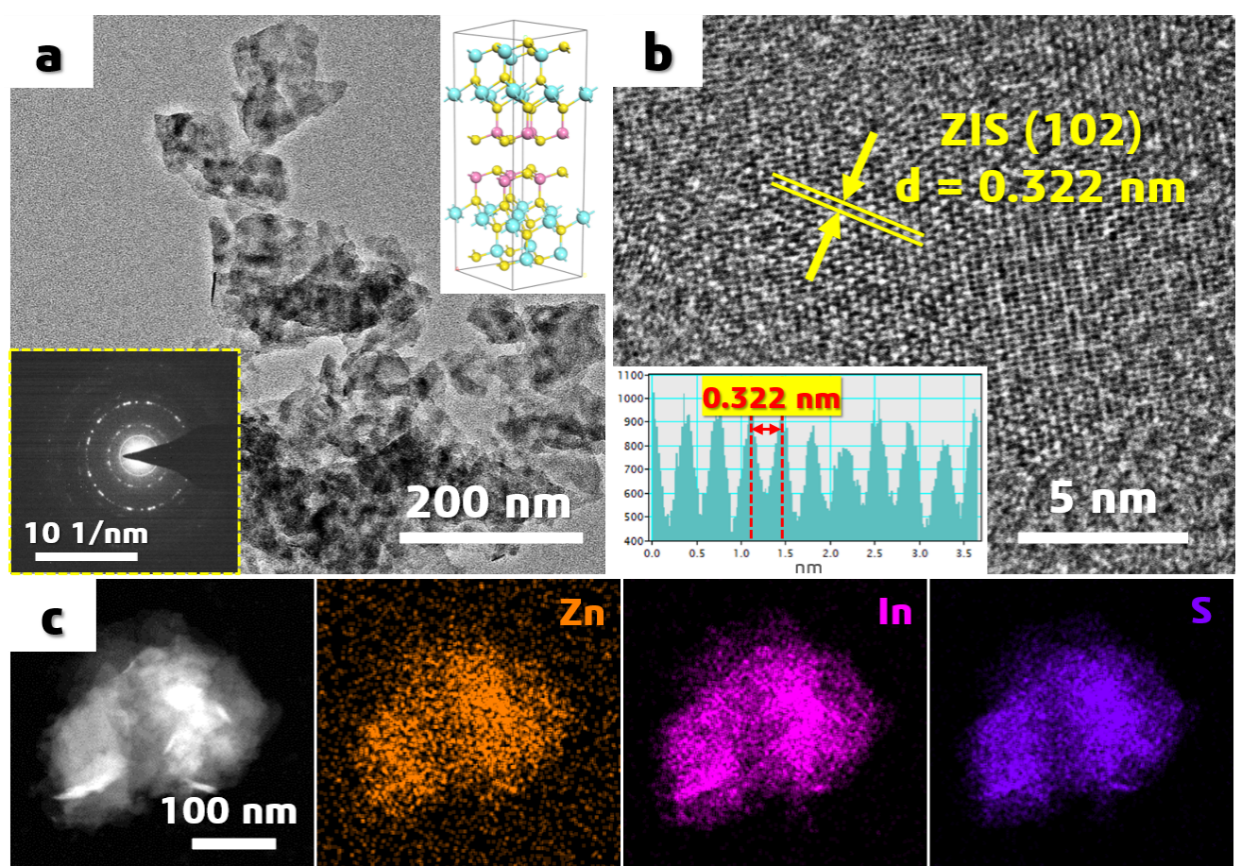


**Figure S5.** (a) TEM image (inset: SAED pattern and structural model of the layered ZnIn_2_S_4_), (b) HRTEM image (inset: fourier transform image) and (c) EDS results of ZIS nanosheets with elemental mapping results.


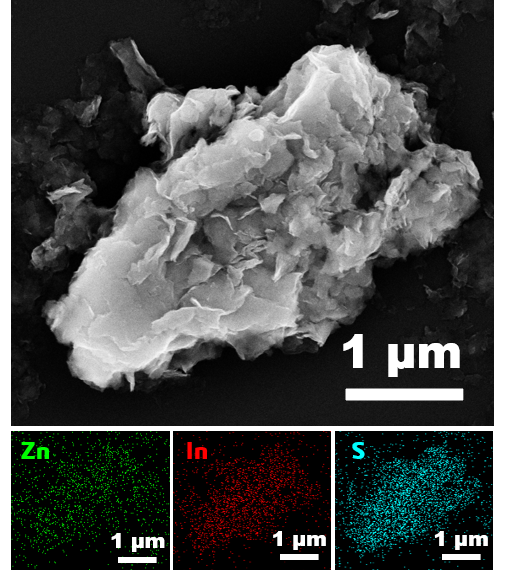


**Figure S6.** SEM image and EDS results of ZIS nanosheets with elemental mapping result.


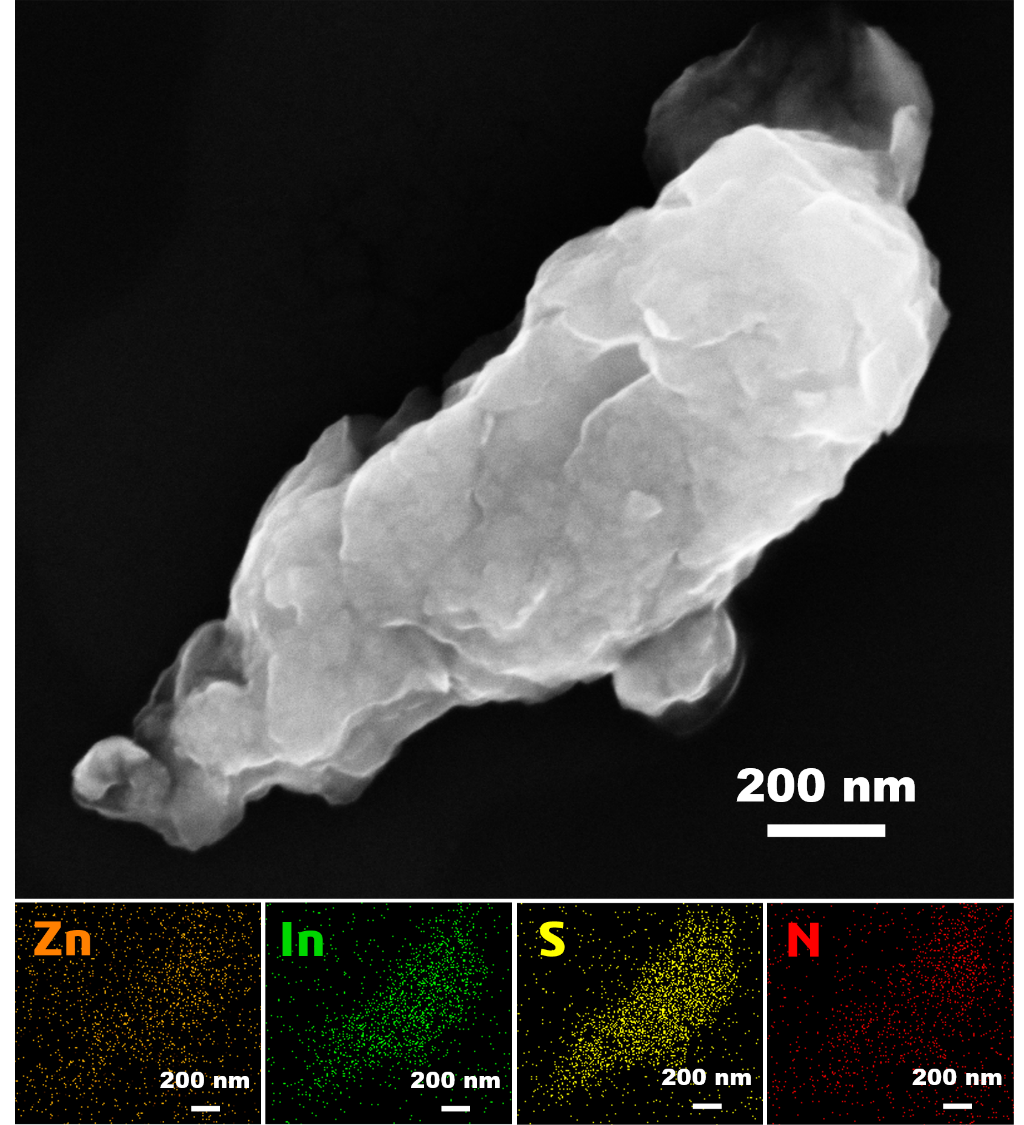


**Figure S7.** SEM image and EDS results of ZIS@10PDDA with elemental mapping result.





**Figure S8.** Time course of 4-NA photoreduction over pure ZIS and ZIS@xPDDA (x=5, 10, 15, 30, 60 mg/mL).





**Figure S9.** Photoactivities of blank ZIS and ZIS@10PDDA toward selective reduction of aromatic nitro compounds, including (a) 4-NA, (b) 2-NA, (c) 3-NA, (d) NB, (e) 2-NP, (f) 3-NP, (g) 4-NP, (h) 4-nitrotoluene, (i) 4-nitroanisole and (j) 4-nitroacetophenone with the addition of ammonium formate as a hole scavenger and N_2_ bubbling at ambient conditions under visible light irradiation along with (k) photocatalytic mechanism of hydrogenation of nitroaromatics. [[^9^](#_ENREF_9)]

**
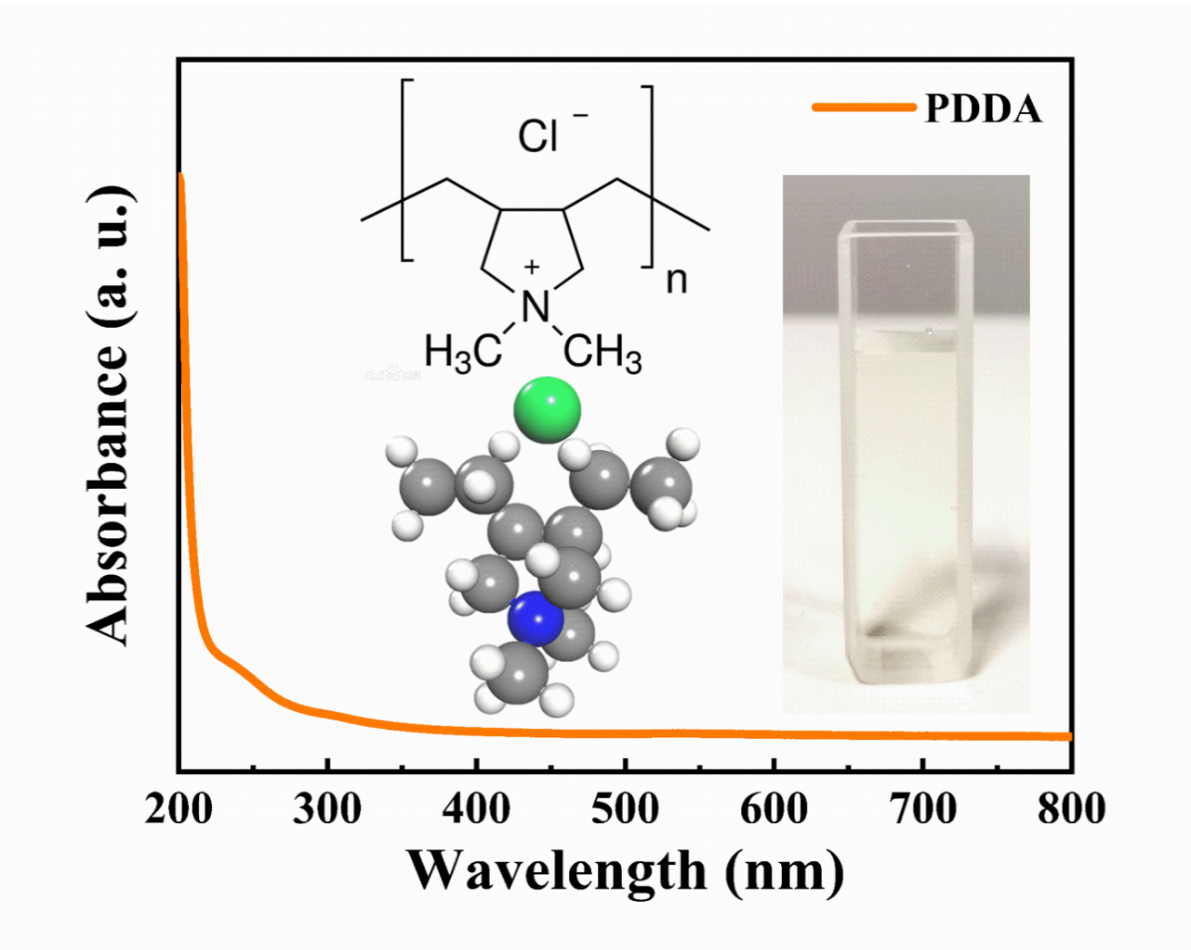
**

**Figure S10.** UV-vis absorption spectrum of PDDA aqueous solution with photograph and molecular structure in the inset.





**Figure S11.** Photocatalytic reduction of 4-NA utilizing pure PDDA under visible light irradiation.





**Figure S12.** Photoactivities of CIS & CIS@10PDDA, In_2_S_3_ & In_2_S_3_@10PDDA, and CdS & CdS@10PDDA toward photoreduction of 4-NA under visible light irradiation (λ > 420 nm).





**Figure S13.** XRD patterns of (a) CIS & CIS@10PDDA, (c) In_2_S_3_ & In_2_S_3_@10PDDA, and (e) CdS & CdS@10PDDA; DRS spectra and Tauc plots of (αhν)^2^ *vs.* hν for (b) CIS & CIS@10PDDA, (d) In_2_S_3_ & In_2_S_3_@10PDDA, and (f) CdS & CdS@10PDDA.

**Note:** XRD pattern of CdIn_2_S_4_ (**Figure S13a)** shows the high-purity cubic phase (JCPDS No. 27-0060) [[^10^](#_ENREF_11)] and CIS@10PDDA heterostructure demonstrates the similar result. As displayed in **Figure S13b**, CIS and CIS@10PDDA heterostructure exhibit the similar absorption edge, indicating PDDA coating does not influence the optical property of CdIn_2_S_4_. Similar results are found in cubic phase In_2_S_3_ (JCPDS No. 65-0459)[[^11^](#_ENREF_12)] and hexagonal phase CdS (JCPDS No. 77-2306)[[^1^](#_ENREF_13)], indicating the PDDA decorated not change the crystal structure and optical properties of TMCs.


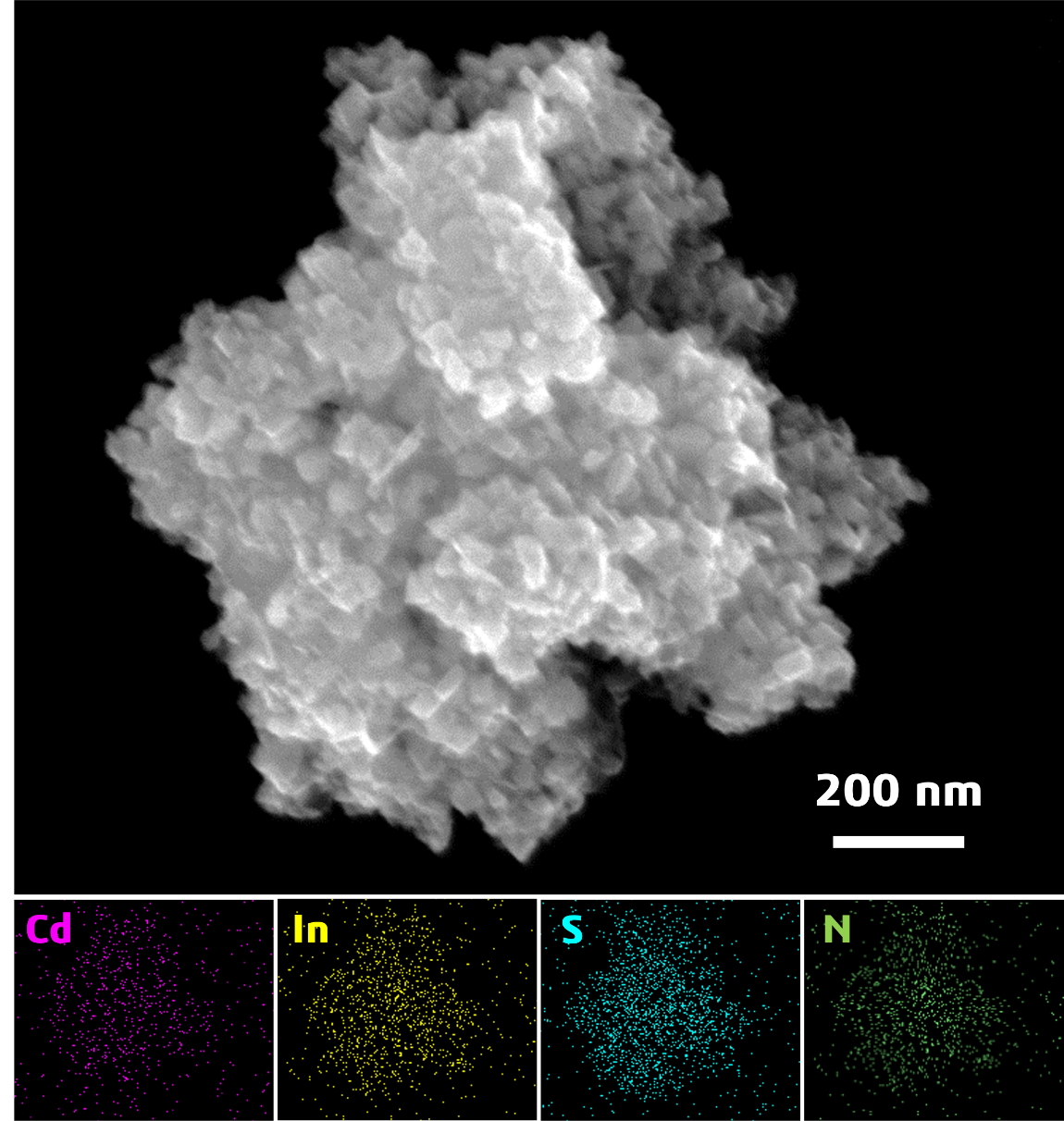


**Figure S14.** SEM images of CIS@10PDDA with elemental mapping result.


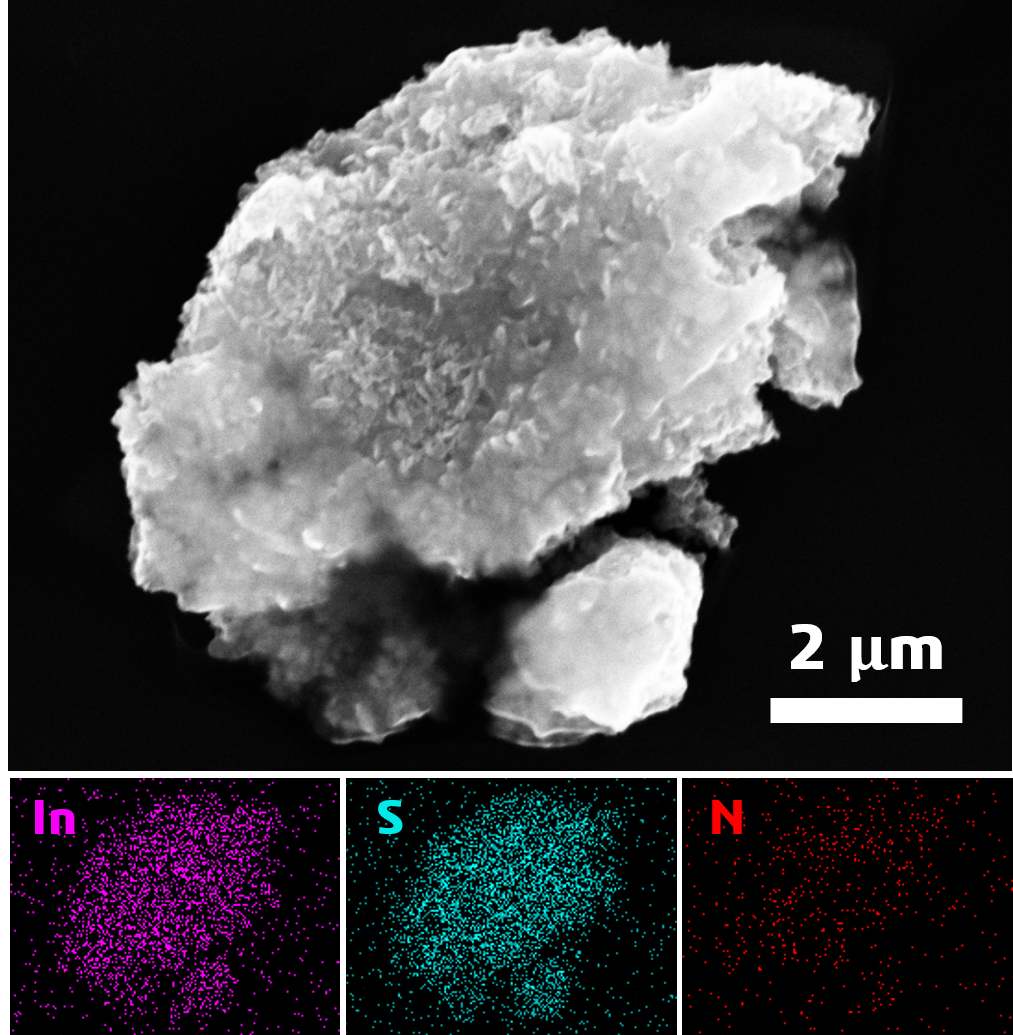


**Figure S15.** SEM images of In_2_S_3_@10PDDA with elemental mapping result.


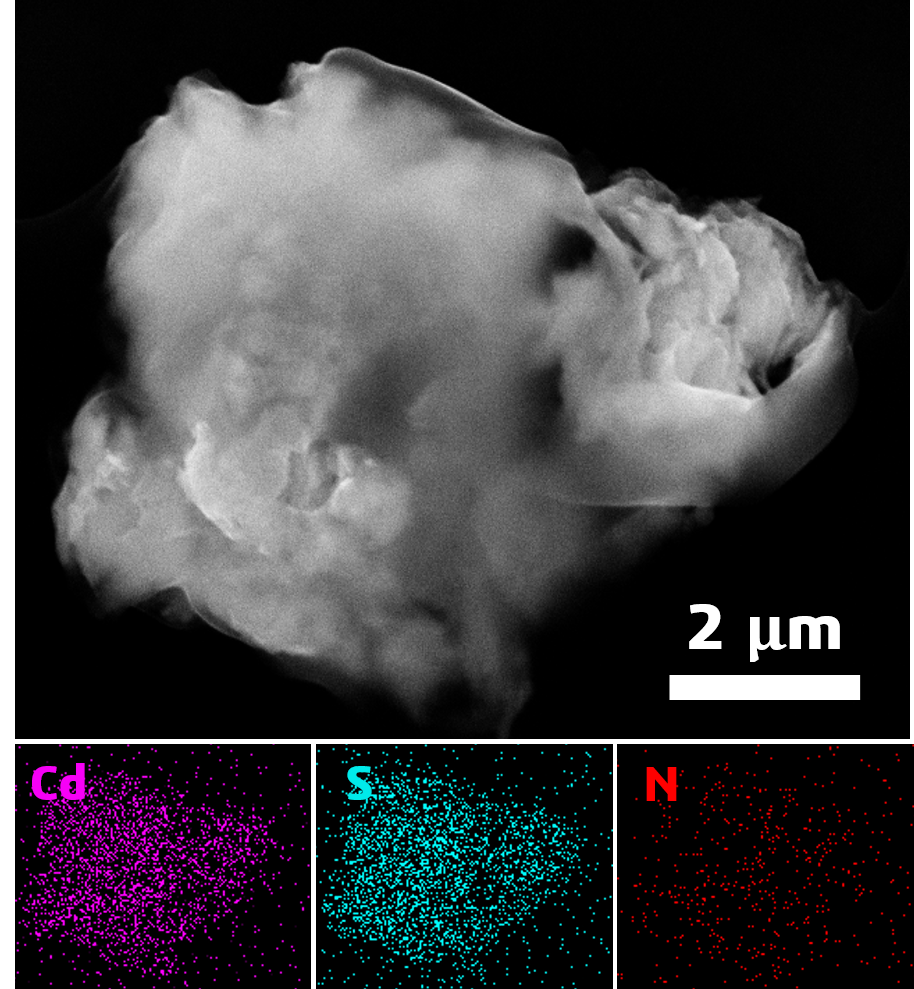


**Figure S16.** SEM images of CdS@10PDDA with elemental mapping result.





**Figure S17.** Photoactivities of ZIS and (a) ZIS@P, (b) ZIS@MP, (c) ZIS@TMAB and (d) ZIS@BMPC toward photoreduction of 4-NA under visible light irradiation (λ > 420 nm).





**Figure S18.** XRD patterns of ZIS@P, ZIS@MP, ZIS@TMAB and ZIS@BMPC.





**Figure S19.** DRS results with Tauc plots of (αhν)^2^ *vs.* light energy for (a) ZIS@P, (b) ZIS@MP, (c) ZIS@TMAB and (d) ZIS@BMPC.

**Note:** As shown in **Figure S19**, ZIS@P, ZIS@MP, ZIS@TMAB and ZIS@BMPC exhibit the similar absorption intensity and edge position to those of blank ZIS substrate, indicating that these amine-containing organic molecules coating fails to influence the optical property of ZIS substrate.





**Figure S20.** (a) Blank experiments for photocatalytic reduction of 4-NA without light irradiation or catalyst. (b) Photoactivities of ZIS@10PDDA with and without adding K_2_S_2_O_8_. Photoactivities of pure ZIS, ZIS+10PDDA and ZIS@10PDDA (c) without adding hole scavenger, and (d) with adding NH_4_COOH as hole scavenger towards 4-NA reduction under visible light irradiation.





**Figure S21.** Photoactivities of ZIS@10PDDA, and ZIS@10PDDA calcined at different temperature (200 ℃, 300 ℃, 400 ℃) towards 4-NA reduction under visible light irradiation.





**Figure S22.** TGA results of ZIS and ZIS@10PDDA.

**Note:** Thermogravimetric analysis (TGA) of ZIS and ZIS@10PDDA was carried out to monitor the thermal stability and degradation of the samples under Ar atmosphere. As shown in **Figure S22**, ZIS shows similar weight loss trend to that of ZIS@10PDDA in the region of 50-250 ℃, corresponding to the release of physically absorbed water. From 250 °C to 600 °C, the difference in weight loss between ZIS (3.15%) and ZIS@10PDDA (5.66%) suggests the gradual removal of bPEI molecules capped on the ZIS surface upon elevating the temperature, thus resulting in about 2.5 % relative weight loss for ZIS@10PDDA.

1



**Figure S23.** XRD patterns of ZIS@10PDDA-200℃, ZIS@10PDDA-300 ℃ and ZIS@10PDDA-400℃.





**Figure S24.** DRS result with corresponding Tauc plot of (αhν)^2^ *vs.* hν for ZIS@10PDDA-200℃.


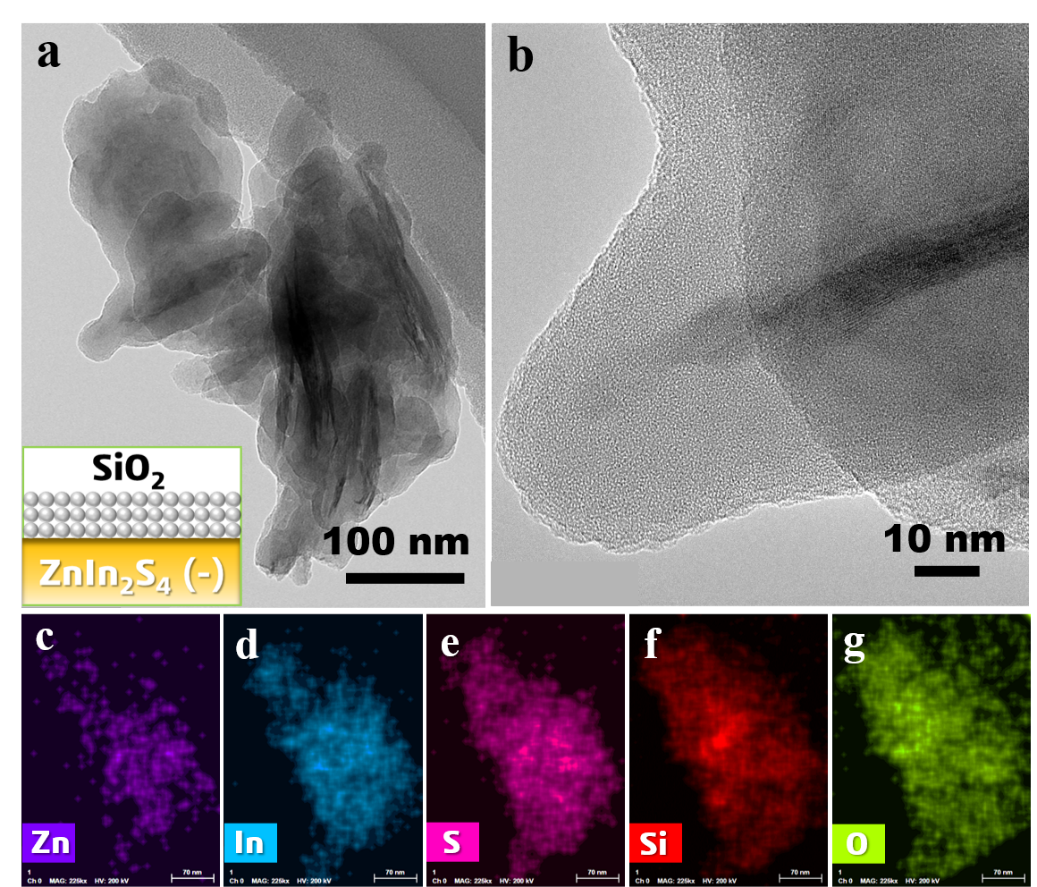


**Figure S25.** (a) TEM and (b) HRTEM images of ZIS@SiO_2_ nanocomposite with (c-g) elemental mapping results.


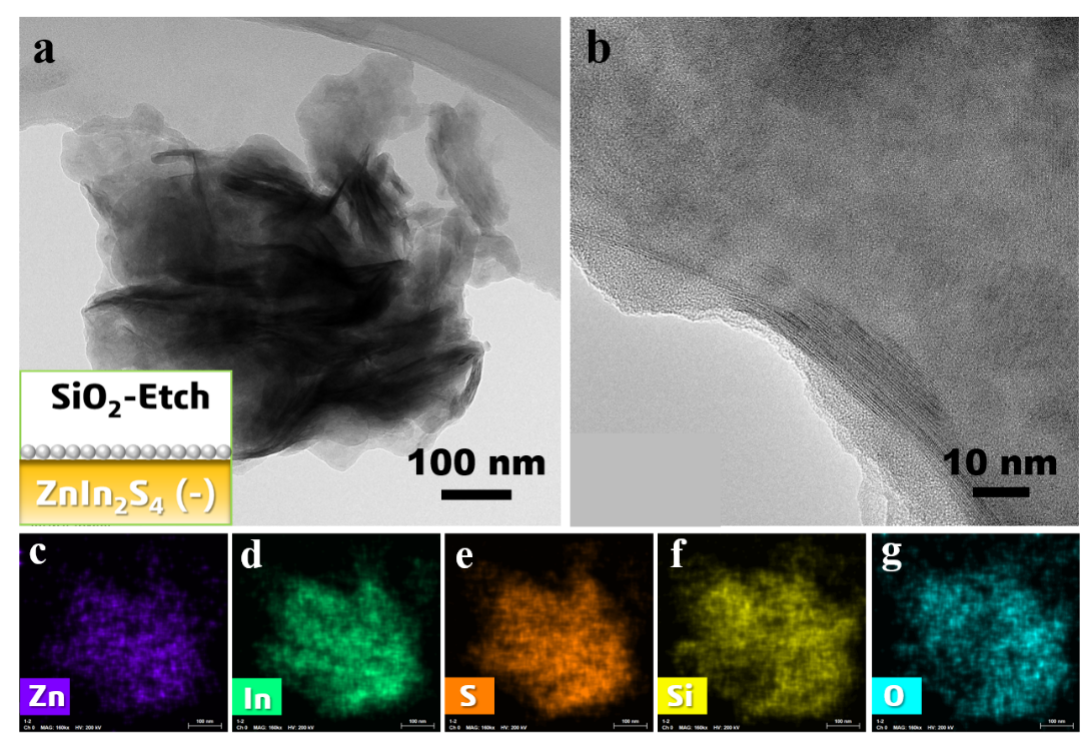


**Figure S26.** (a) TEM and (b) HRTEM images of ZIS@SiO_2_-Etch nanocomposite with (c-g) elemental mapping results.


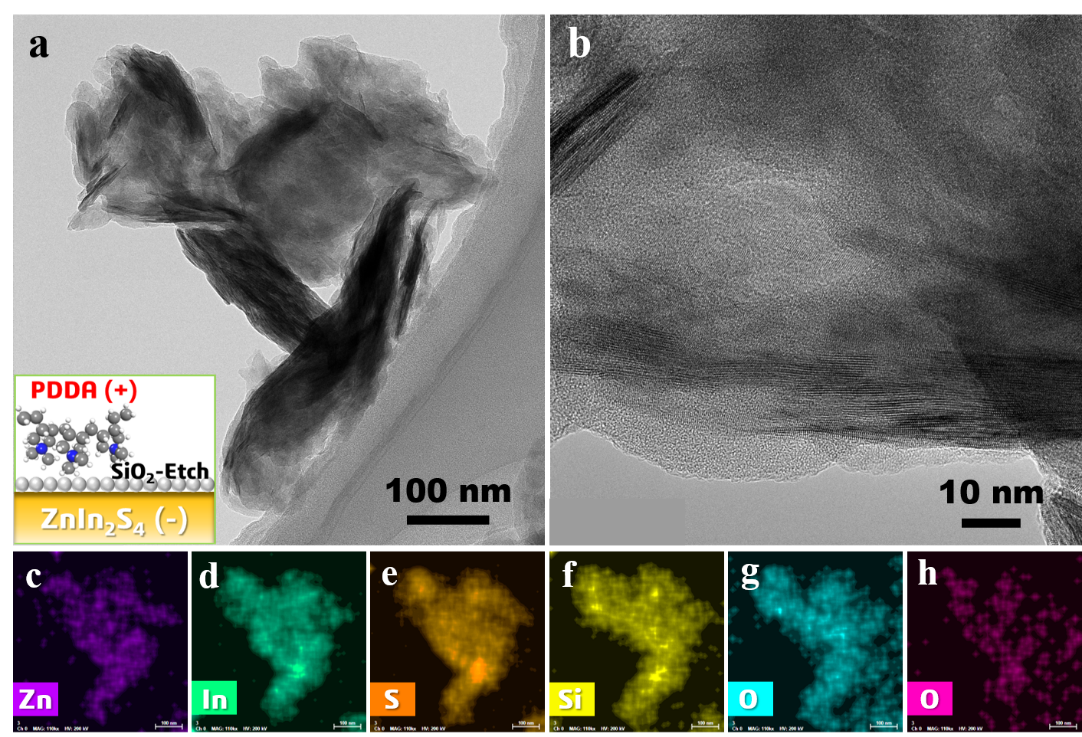


**Figure S27.** (a) TEM and (b) HRTEM images of ZIS@SiO_2_-Etch@10PDDA nanocomposite with (c-g) elemental mapping results.





**Figure S28.** Cyclic reaction of ZIS and ZIS@10PDDA toward photoreduction of 4-NA under visible light irradiation.





**Figure S29.** XRD pattern of ZIS@10PDDA after 5 cycles.





**Figure S30.** High-resolution (a) Zn 2p, (b) In 3d, (c) S 2p, and (d) N1s spectra of ZIS@10PDDA after 5 cyclic reactions.

**Note:** In the high-resolution Zn 2p spectrum, two peaks with the binding energies of 1044.25 and 1021.21 eV correspond to the Zn(II) oxidation state.[[^11^](#_ENREF_12)] High-resolution In 3d spectrum with two peaks at 444.78 and 452.35 eV correspond to the In cation with a valence state of +3.[[^11^](#_ENREF_12)] The peaks at 161.41 and 162.68 eV in the high-resolution S 2p spectrum are assigned to the divalent sulfide ions (S^2-^),[[^12^](#_ENREF_14)] which is in line with the elemental chemical states of ZnIn_2_S_4_. The two peaks in the high-resolution N 1s spectrum at 399.72 and 402.44 eV are attributed to the absorbed N and quaternary amines.[[^13^](#_ENREF_15)] In general, XPS results of ZIS@10PDDA after 5 cyclic reactions are in faithful agreement with those of pristine counterpart.


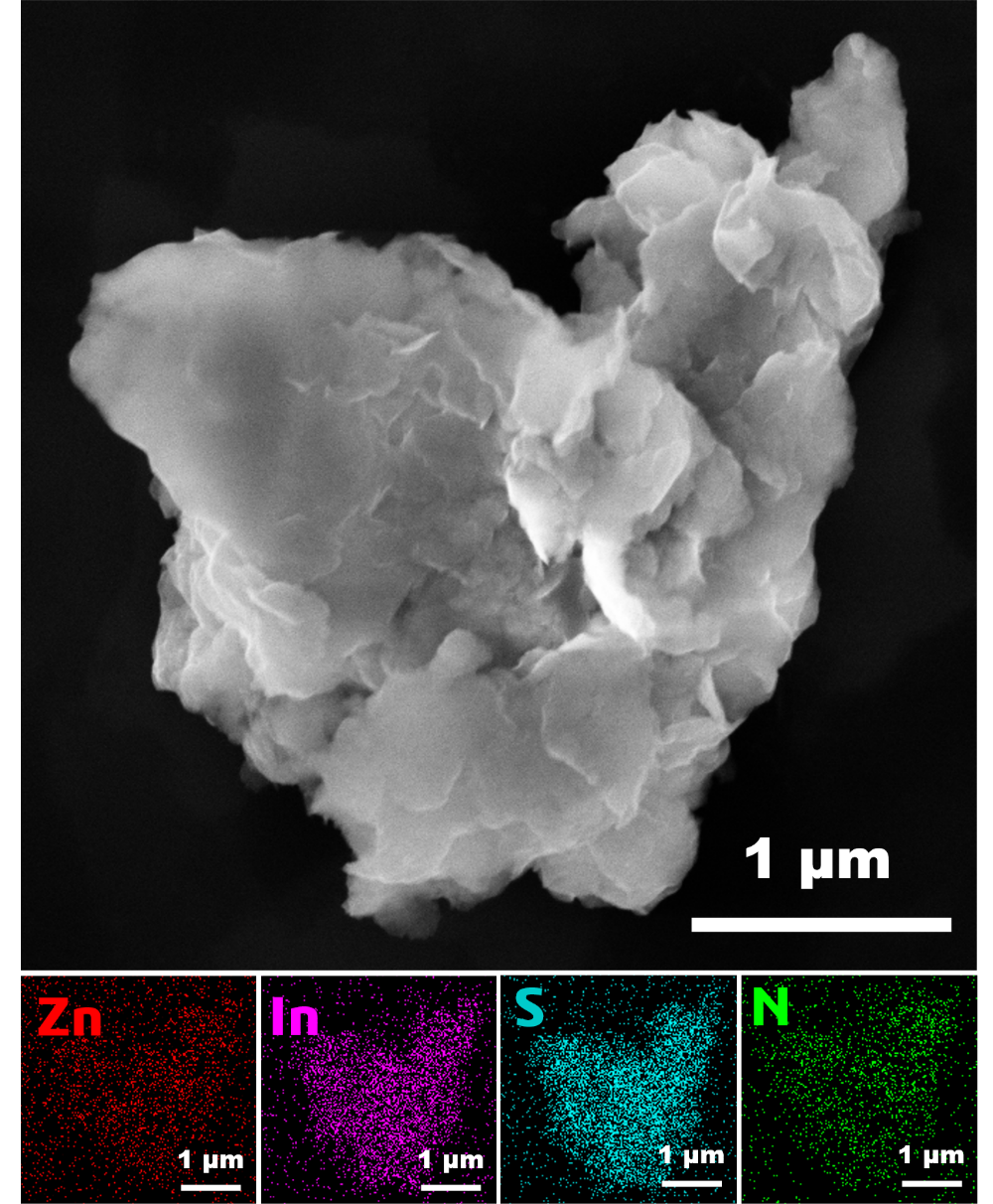


**Figure S31.** SEM image and EDS results of ZIS@10PDDA after 5 cycles with elemental mapping result.





**Figure S32.** Photoactivities of ZIS@10PDDA under different light intensity.





**Figure S33.**  Photoactivities of ZIS@10PDDA in the presence of different sacrificial reagents.





**Figure S34.**  Wavelength-dependent CO_2_ photoreduction activities of ZIS@10PDDA.





**Figure S35.** Cyclic reaction of ZIS@10PDDA under continuous visible light irradiation ($\lambda$ > 420 nm) for 10 h.





**Figure S36.** (a) Nyquist plots of electrochemical impedance spectroscopy (EIS) for ZIS and ZIS@10PDDA under visible light irradiation (λ>420 nm); (b) Open-circuit-potential and average electron lifetime (τ_n_) (inset) of ZIS and ZIS@10PDDA calculated based on the OCVD results; (c) schematic illustration of the PEC water dissociation mechanism.

**Note:** Average electron lifetime (τ_n_) of the semiconductor photoelectrodes can be determined by the following equation

*τ* =*k_B_T*/e(*dV_oc_/dt*)^-1^ *(5)*

where *τ* is the potential-dependent photoelectron lifetime, *k_B_* is the Boltzmann’s constant (1.38 x 10^-23^ J/K), *T* is the temperature (298K), *e* is the charge of a single electron (1.6 x10^-19^ C), and *V_oc_* is the open-circuit voltage at time *t*.[[^14^](#_ENREF_17)]


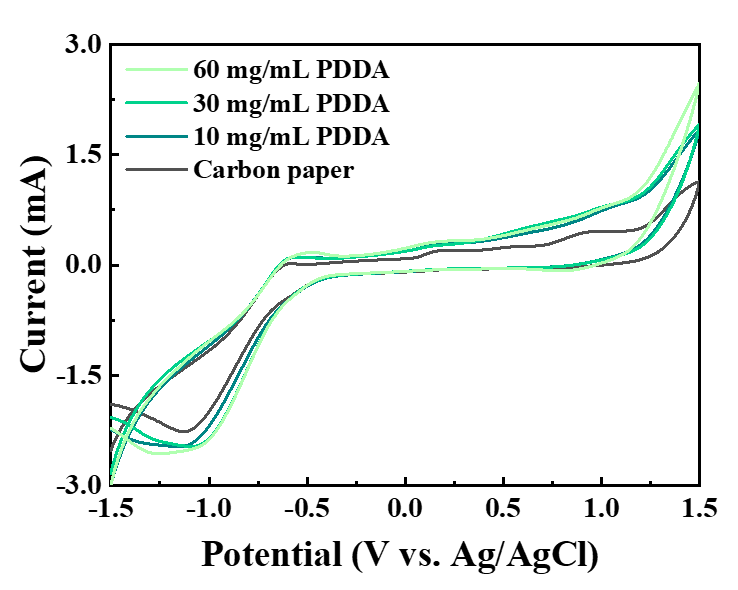


**Figure S37.** CV curves of PDDA with different concentration (10, 30, 60 mg/mL) in CH_2_Cl_2_ with 0.1 M tetrabutylammonium tetrafluoroborate for scanning range of -1.5~1.5 V.


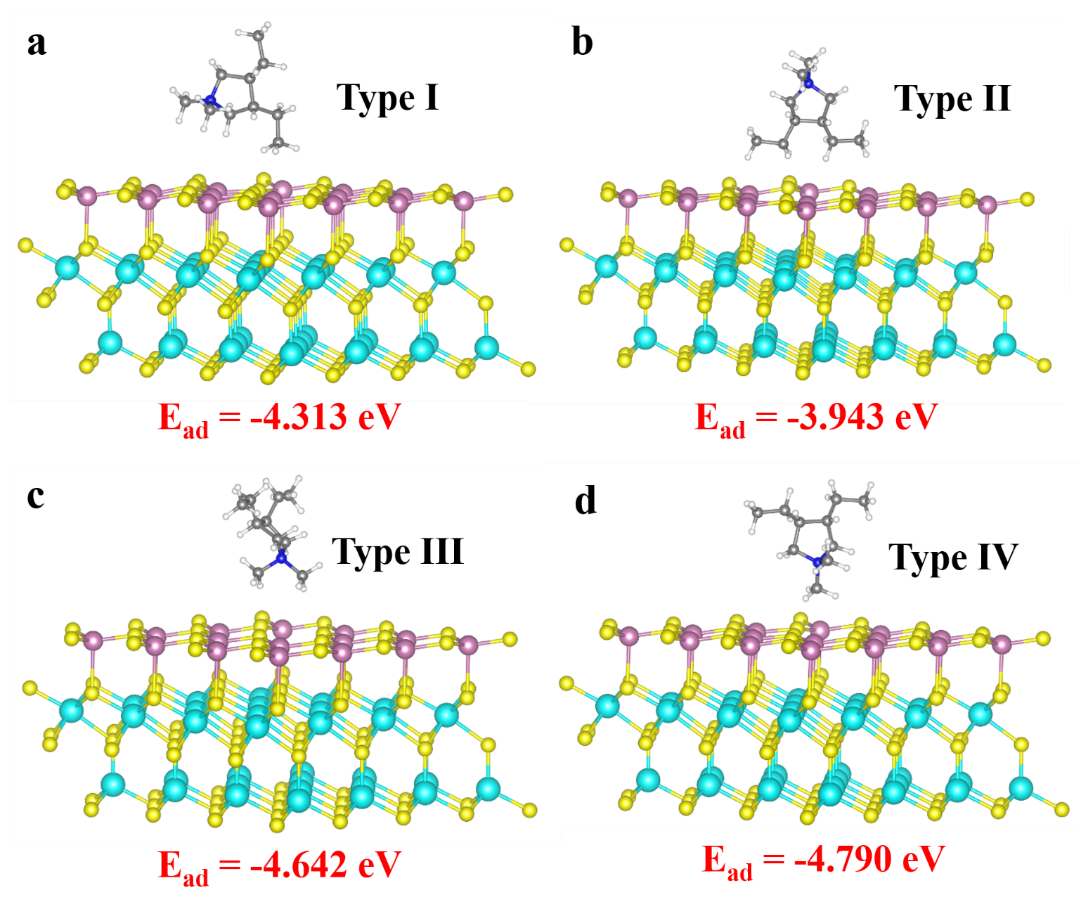


**Figure S38.** Different absorption configurations of PDDA on ZIS surface with Zn defect.


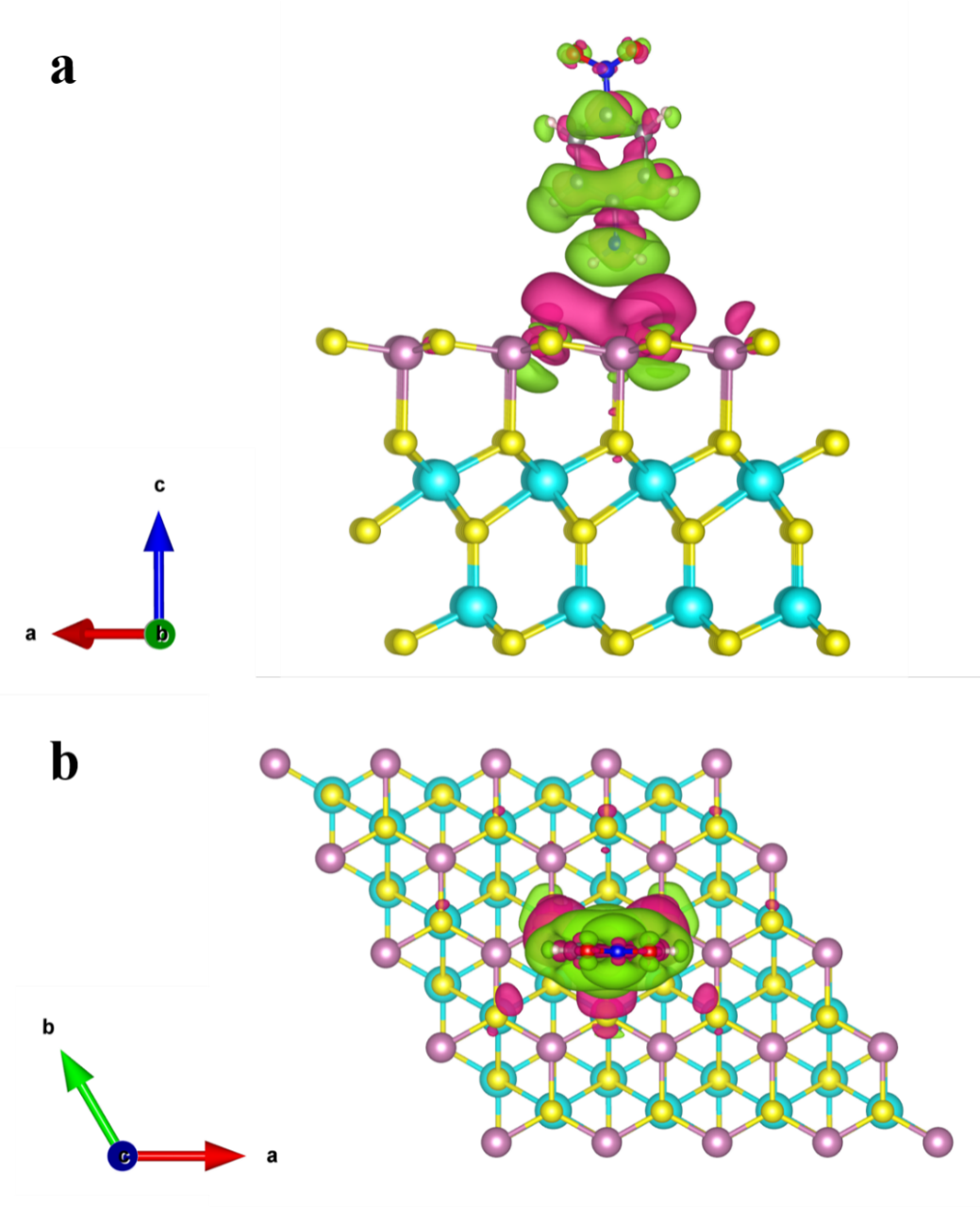


**Figure S39.** Plot of charge density difference (green: charge depletion, violet: charge accumulation) for the most energy favorable model of 4-NA molecular absorbed on the ZnIn_2_S_4_ monolayer surface. The isosurface of charge density is set to be 0.0003 e/Å^3^.


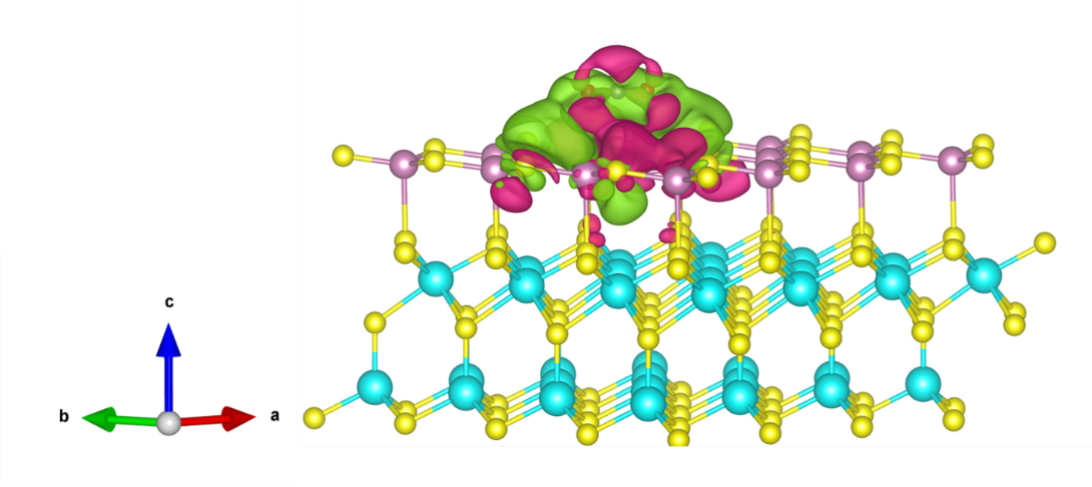


**Figure S40.** Plot of charge density difference (green: charge depletion, violet: charge accumulation) for the most energy favorable model of CO_2_ molecular absorbed on the ZnIn_2_S_4_ monolayer surface. The isosurface of charge density is set to be 0.0004 e/Å^3^.





**Figure S41** Mott–Schottky plots of (a) ZIS and (b) ZIS@10PDDA.

**Table S1.** Peak position with corresponding functional groups.

| **Peak position (cm^-1^)** | **Chemical bond** | **Reference** |
| --- | --- | --- |
| 3450 | Surface adsorbed water | [[^15^](#_ENREF_18)] |
| 2914, 2858 | -CH_2_ | [[^16^](#_ENREF_19)] |
| 1624 | -OH | [[^17^](#_ENREF_20)] |
| 1466 | -C-N- | [[^18^](#_ENREF_21)] |
| 1130 | -C-N- | [[^19^](#_ENREF_22)] |

**Table S2.** Specific surface area, pore volume and pore size of ZIS and ZIS@10PDDA heterostructure.

| ***Sample*** | ***S_BET_***  ***(******m^2^/g)*** | ***Total pore volume***  ***(cm^3^/g)*** | ***Average pore size***  ***(nm)*** |
| --- | --- | --- | --- |
| ZIS | 19.44 | 0.034 | 4.44 |
| ZIS@10PDDA | 1.89 | 0.011 | 12.87 |

**^a^** BET surface area is calculated from the linear part of BET plots.

**^b^** Single point total pore volume of the pores at P/P0 = 0.99.

**^c^** Adsorption average pore width (4V/A by BET).

**Table S3.** Chemical bond species vs. B.E. for different samples.

| ***Elements*** | ***ZIS*** | ***PDDA*** | ***ZIS@10PDDA*** | ***ZIS@10PDDA***  ***after reaction*** | ***Chemical bond species*** | ***Ref.*** |
| --- | --- | --- | --- | --- | --- | --- |
| Zn 2p_3/2_ | 1021.58 | N.D. | 1021.14 | 1021.21 | Zn^2+^ | [[^11^](#_ENREF_12)] |
| Zn 2p_1/2_ | 1044.56 | N.D. | 1044.15 | 1044.25 | Zn^2+^ |
| In 3d_5/2_ | 445.25 | N.D. | 444.68 | 444.78 | In^3+^ | [[^11^](#_ENREF_12)] |
| In 3d_3/2_ | 452.79 | N.D. | 452.22 | 452.35 | In^3+^ |
| S 2p_3/2_ | 161.81 | N.D. | 161.31 | 161.41 | S^2-^ | [[^12^](#_ENREF_14)] |
| S 2p_1/2_ | 163.15 | N.D. | 162.66 | 162.68 | S^2-^ |
| C 1s | 284.80 | 284.80 | 284.80 | 284.80 | C-C | [[^20^](#_ENREF_23)], [[^21^](#_ENREF_24)] |
| C 1s | N.D. | 286.06 | 286.00 | 286.02 | C-N |
| C 1s | 286.55 | N.D. | 286.69 | 286.71 | C-O |
| C 1s | 287.51 | N.D. | 287.38 | 287.92 | C=O |
| N 1s | N.D. | 399.71 | 399.71 | 399.72 | Adsorbed N | [[^13b^](#_ENREF_16), [^22^](#_ENREF_25)] |
| N 1s | N.D. | 402.23 | 402.4 | 402.44 | Quaternary amine |

N.D.: Not Detected

**Table S4**. Fitted EIS results of different photoanodes based on the equivalent circuit.

| **Sample** | **R_s_ /ohm** | **R_ct_ /ohm** | **CPE/(F·cm^-2^)** |
| --- | --- | --- | --- |
| ZIS | 12.59 | 6514 | 8.004×10^-5^ |
| ZIS@10PDDA | 13.98 | 5434 | 6.74×10^-5^ |

**Table S5.** Computed adsorption energy of ZnIn_2_S_4_ and ZnIn_2_S_4_@bPEI for the optimized configurations of H_2_O and 4-NA absorbed at different positions.

| ***Position*** | ***4-NA*** | | ***CO_2_*** | |
| --- | --- | --- | --- | --- |
|  | ***ZnIn_2_S_4_*** | ***ZnIn_2_S_4_@PDDA*** | ***ZnIn_2_S_4_*** | ***ZnIn_2_S_4_@PDDA*** |
| Top of Zn | **-0.292 eV** | 0.205 | 0.189 | 0.665 |
| Middle of Zn-S | -0.285 eV | **-0.320** | 0.093 | 0.095 |
| Top of S | -0.215 eV | -0.168 | **-0.009** | 0.095 |
| Top of PDDA | - | -0.093 | - | **-0.045** |

**References**

[1] X. Xie, N. Zhang, Z.-R. Tang, M. Anpo, Y.-J. Xu, *Appl. Catal. B: Environ.* **2018**, 237, 43.

[2] C. Yuan, T. Wu, J. Mao, T. Chen, Y. Li, M. Li, Y. Xu, B. Zeng, W. Luo, L. Yu, G. Zheng, L. Dai, *J. Am. Chem. Soc.* **2018**, 140, 7629.

[3] J. Hafner, *J. Comput. Chem.* **2008**, 29, 2044.

[4] G. Wang, L. Peng, K. Li, L. Zhu, J. Zhou, N. Miao, Z. Sun, *Comput. Mater. Sci.* **2021**, 186, 110064.

[5] P. E. Blochl, *Phys. Rev. B* **1994**, 50, 17953.

[6] J. P. Perdew, Y. Wang, *Phys. Rev. B* **1992**, 45, 13244.

[7] J. P. Perdew, K. Burke, M. Ernzerhof, *Phys. Rev. Lett.* **1997**, 78, 1396.

[8] a) S. Grimme, S. Ehrlich, L. Goerigk, *J. Comput. Chem.* **2011**, 32, 1456; b) S. Grimme, J. Antony, S. Ehrlich, H. Krieg, *J. Chem. Phys.* **2010**, 132, 154104.

[9] a) L. Hu, X. Cao, D. Ge, H. Hong, Z. Guo, L. Chen, X. Sun, J. Tang, J. Zheng, J. Lu, H. Gu, *Chemistry* **2011**, 17, 14283; b) R. Millán, L. Liu, M. Boronat, A. Corma, *J. Catal.* **2018**, 364, 19.

[10] Q. Zhang, J. Wang, X. Ye, Z. Hui, L. Ye, X. Wang, S. Chen, *ACS Appl. Mater. Interfaces* **2019**, 11, 46735.

[11] M. Q. Yang, Y. J. Xu, W. Lu, K. Zeng, H. Zhu, Q. H. Xu, G. W. Ho, *Nat. Commun.* **2017**, 8, 14224.

[12] S. Zhang, X. Liu, C. Liu, S. Luo, L. Wang, T. Cai, Y. Zeng, J. Yuan, W. Dong, Y. Pei, Y. Liu, *ACS Nano* **2018**, 12, 751.

[13] a) R. Xia, S. Zhang, X. Ma, F. Jiao, *J. Mater. Chem. A* **2020**, 8, 15884; b) S. Wang, X. Wang, S. P. Jiang, *Phys. Chem. Chem. Phys.* **2011**, 13, 6883.

[14] S. Hou, X. C. Dai, Y. B. Li, M. H. Huang, T. Li, Z. Q. Wei, Y. H. He, G. C. Xiao, F. X. Xiao, *J. Mater. Chem. A* **2019**, 7, 22487.

[15] H. Yang, R. Cao, P. Sun, J. Yin, S. Zhang, X. Xu, *Appl. Catal. B: Environ* **2019**, 256, 117862.

[16] M. R. Nabid, Y. Bide, M. Shojaipour, F. Dastar, *Catal. Lett* **2015**, 146, 229.

[17] B. Chai, T. Peng, P. Zeng, X. Zhang, *Dalton Trans.* **2012**, 41, 1179.

[18] X. Zeng, Y. Liu, Y. Kang, Q. Li, Y. Xia, Y. Zhu, H. Hou, M. H. Uddin, T. R. Gengenbach, D. Xia, C. Sun, D. T. McCarthy, A. Deletic, J. Yu, X. Zhang, *ACS Catal.* **2020**, 10, 3697.

[19] F. Gao, X. Du, X. Hao, S. Li, X. An, M. Liu, N. Han, T. Wang, G. Guan, *Chem. Eng. J.* **2017**, 328, 293.

[20] L. Ye, J. Fu, Z. Xu, R. Yuan, Z. Li, *ACS Appl. Mater. Interfaces* **2014**, 6, 3483.

[21] C. S. Mo, J. H. Jian, J. Li, Z. S. Fang, Z. Zhao, Z. K. Yuan, M. J. Yang, Y. Zhang, L. M. Dai, D. S. Yu, *Energy Environ. Sci.* **2018**, 11, 3334.

[22] W.-B. Tseng, C.-H. Lee, W.-L. Tseng, *ACS Appl. Nano Mater.* **2018**, 1, 6808.
